# Dexmedetomidine reduces propofol-induced hippocampal neuron injury by modulating the miR-377-5p/Arc pathway

**DOI:** 10.1186/s40360-022-00555-9

**Published:** 2022-03-25

**Authors:** Zong Chen, Yong Ding, Ying Zeng, Xue-Ping Zhang, Jian-Yan Chen

**Affiliations:** 1 Department of Anesthesiology, The First Affiliated Hospital of Guangdong Pharmaceutical University, NO.19 Nonglin Road, Yuexiu District, Guangzhou, Guangdong Province China; 2 Department of Anesthesiology, Shenzhen Shajin Hospital Affiliated to Guangzhou Medical University, Shenzhen, China; 3 Department of Anesthesiology, Shenzhen People’s Hospital, Shenzhen Anesthesiology Engineering Center, The Second Clinical Medical College of Jinan University, NO. 1017 Dongmen North Road, Luohu District, Shenzhen, Guangdong Province China

**Keywords:** Dexmedetomidine, Propofol, DNMT3A, Methylation, Neuroprotection, Neurotoxicity, Apoptosis, Viability

## Abstract

**Background:**

Propofol and dexmedetomidine (DEX) are widely used in general anesthesia, and exert toxic and protective effects on hippocampal neurons, respectively. The study sought to investigate the molecular mechanisms of DEX-mediated neuroprotection against propofol-induced hippocampal neuron injury in mouse brains.

**Methods:**

Hippocampal neurons of mice and HT22 cells were treated with propofol, DEX, and propofol+DEX. In addition, transfection of miR-377-5p mimics or inhibitors was performed in HT22 cells. Neuronal apoptosis was evaluated by a means of terminal deoxynucleotidyl transferase (TdT)-mediated dUTP nick end labeling (TUNEL) or Hochest 33,258 staining; Arc positive expression in hippocampus tissues was detected using a microscope in immunohistochemistry assays; miRNA-377-5p expression was quantified by RT-qPCR; the protein levels of Arc, DNMT3A, and DNMT3B were determined using western blot; Cell Counting Kit-8 (CCK-8) assay was used to detect the viability and apoptotic rate of the neurons; methylation analysis in the miR-377-5p promoter was performed through methylated DNA immunoprecipitation (MeDIP) assay; dual luciferase reporter assay was performed to confirm whether Arc was under targeted regulation of miR-377-5p.

**Results:**

In the current study, both *in vitro* and *in vivo*, propofol treatment induced hippocampal neuron apoptosis and suppressed cell viability. DNMT3A and DNMT3B expression levels were decreased following propofol treatment, resulting in lowered methylation in the miR-377-5p promoter region and then enhanced expression of miR-377-5p, leading to a decrease in the expression of downstream Arc. Conversely, the expression levels of DNMT3A and DNMT3B were increased following DEX treatment, thus methylation in miR-377-5p promoter region was improved, and miR-377-5p expression was decreased, leading to an increase in the expression of downstream Arc. Eventually, DEX pretreatment protected hippocampal neurons against propofol-induced neurotoxicity by recovering the expression levels of DNMT3A, miR-377-5p, and Arc to the normal levels. Additionally, DNMT3A knockdown improved miR-377-5p expression but reduced Arc expression, and DNMT3A overexpression exerted the opposite effects. Dual luciferase reporter assay revealed a binding target between miR-377-5p and Arc 3’UTR. The neuroprotective effect of DEX against propofol-induced neuronal apoptosis was diminished after Arc knockdown. Silencing Arc independently triggered the apoptosis of HT22 cells, which was alleviated through transfection of miR-377-5p inhibitors.

**Conclusions:**

DEX reduced propofol-induced hippocampal neuron injury via the miR-377-5p/Arc signaling pathway.

**Supplementary Information:**

The online version contains supplementary material available at 10.1186/s40360-022-00555-9.

## Background

The brain is an organ processing and storing information from the outside circumstance, and memories refer to the information storage through neuronal synaptic connections in the brain. Investigating the molecular mechanism of memory remains a huge challenge in the modern neuroscience, due to the complexity of brain structures. The “engram” was a hypothetical molecular basis of the memory, and theorized that the memory was encoded by some neuronal ensembles sparsely distributed in neural circuits [1]. The cellular and molecular mechanisms of the “engram” consisted of synaptic changes and modulation of gene expression [2, 3].

Some genes are involved in memory formation, such as *BDNF* (brain derived neurotrophic factor) [4], *REELIN* [5], N-methyl d-aspartate (NMDA) receptor subunit *NR1* [6], NMDA receptor subunit *NR3B* [7], *PPP3CA* (protein phosphatase 3 catalytic subunit alpha) [8], *METTL3* (methyltransferase like 3) [9], CREB (cAMP responsive element binding) [10], *Arc* (activity-regulated cytoskeletal), *Egr1* (early growth response 1) [11], etc. Among these genes, *Arc* and *Egr1* belong to immediate early genes (IEGs), which have been widely used as direct molecular markers to measure neuronal activity for decades, owing to dynamical change in the expression of IEGs promptly in response to neuronal activity [12–16]. Arc is a synaptic activity-induced effector, and is directly regulated by Egr1, and contributes to modulate the synaptic plasticity associated with learning and memory processing [17].

*Arc* is associated with memory-related behaviors, for instance, singing-driven Arc expression changes with the number of songs produced by juvenile songbirds, rather than circadian rhythm [18]. Previous studies demonstrated that abundant Arc proteins were produced when mouse brains were active or sober [19–23]. For human, *Arc* is critical for regulation of synaptic and neuronal plasticity, including long-term change of synaptic strength (long-term potentiation and depression), synaptic scaling, and long-term memory formation. Owing to implication in memory consolidation and reconsolidation processes, *Arc* plays a crucial role in the learning and long-term memory [24, 25]. Furthermore, *Arc* is associated with some potential memory-related behaviors such as drug addiction, a recent study indicated that *Arc* might contribute to drug addiction due to regulation of drug-taking vulnerability [26–28].

Recently, Chen T, et al. [29, 30] found that *Arc* silence promoted neuronal apoptosis and aggravated neuronal death, leading to exacerbating traumatic brain injury. Moreover, they revealed that elevated expression of Arc was detected after traumatic neuronal injury, suggesting that the endogenous Arc protein served as a potential protective factor. Arc dysregulation was related to cognitive disorders such as alzheimer disease (AD) and autism [31]. In addition, Zeng Q, et al. [32] found that *Arc* knockdown increased hippocampal neuron apoptosis and revoked the beneficial effect of 3’-daidzein sulfonate sodium on cognitive impairment. Conversely, Arc overexpression improved hippocampal neuronal density and reduced learning and memory impairments caused by chronic cerebral hypoperfusion [34].

Arc protein expression was reported to be suppressed by several anesthetics such as propofol [33, 34]. Although low-dose propofol was safe for brain growth spurt, recommended or high-dose propofol promoted hippocampal neuroapoptosis and induced cognitive defects. Thus, reduplicative use of propofol triggered long-term cognitive dysfunction [35]. Whether propofol induces hippocampal neuron injury by decreasing Arc expression is unclear.

Dexmedetomidine (DEX), as a highly selective α2-adrenoceptor agonist, has been proved to have the neuroprotective potential, and is widely used in anesthesia and intensive care setting for sedate patients [36–41]. DEX possesses sedative, anxiolytic, sympatholytic, analgesic, and anesthetic properties [42]. Like propofol, DEX is widely used in general anesthesia [43]. Both propofol and DEX, as nonbenzodiazepine agents, are recommended by guidelines to be first-line medications to provide light sedation [44]. Previous studies revealed that compared with propofol alone, the combination of low-dose DEX and propofol could decrease propofol consumption in patients undergoing sedation for ambulatory colonoscopy or magnetic resonance imaging, without enhancing the incidence of side effects [45–49]. Moreover, the combination of DEX and propofol was reported to minimize respiratory depressive effects and lessen surgery-stimulated physiologic stress-response [50–52]. Actually, low-dose DEX was effective to alleviate emergence delirium after intravenous propofol anesthesia during tonsillectomy [53].

In accordance with GSE106799 dataset, the expression levels of both Arc and DNMT3A (DNA methyltransferase 3 alpha) were decreased while the miR-377-5p expression level was increased following exposure to propofol. Of note, propofol caused 29.0-fold decrease of Arc expression (*P* = 0.00035), suggesting a dramatic impact on Arc-mediated function. There is a CpG island located at -1500 bp in the promoter region of miR-377, suggesting that miR-377-5p expression is under regulation of DNA methylation. miR-377-5p was predicted to target the 3’ untranslated region (3’-UTR) of *Arc*, implying that Arc expression is regulated by miR-377-5p. In this study, our preliminary experiment demonstrated that DEX upregulated the expression of DNMT3A and Arc, but downregulated the expression of miR-377-5p. Therefore, we hypothesized that DEX reversed the inhibitory effect of propofol on Arc by regulating DNMT3A/miR-377-5p, whereby suppressing the neurotoxicity of propofol. This study focused on identifying this hypothesis.

## Methods

### Animals and treatments

All animal experiments performed on live animals were approved by the independent Animal Ethical Committee of the First Affiliated Hospital of Guangdong Pharmaceutical University (Guangdong, China) and adhered to relevant guidelines including the ARRIVE guidelines for animal experiments in the study. C56BL/6 mice (21 ± 3 days) from an inbred colony were provided by the animal department of the Xiangya School of Medicine of Central South University, Changsha, China. The mice were randomly divided into four groups: control group (*n* = 4), propofol group (*n*  = 4, 50 mg/kg), propofol + DEX group (*n* = 4, 50 mg/kg propofol + 100 µg/kg DEX), DEX group (*n *= 4, 100 µg/kg). In the propofol group, the mice were treated with 50 mg/kg of propofol, another 50 mg/kg of propofol was administrated following 60 min of recovery. In the propofol + DEX group, the mice were administrated with 100 µg/kg of DEX and then treated with 50 mg/kg of propofol at an interval of 30 min. Both propofol and DEX were administrated using intraperitoneal injection in all of the groups. All mice were euthanized by decapitation after 4 h of treatments, and the hippocampi were quickly removed from the mouse brains, dissected and immediately frozen in liquid nitrogen. The samples were stored at −80 °C until further study.

### Immunohistochemistry and TUNEL staining

After overnight baking at 60 °C, the thick paraffin sections were deparaffinized, rehydrated, and digested with pepsin. Normal horse or goat serums were used for blocking non-specific binding sites for 20 min. The collagen II or collagen X primary antibody (Beyotime, Shanghai, China) was added and the slide was incubated at 4 °C overnight. On the second day, secondary biotinylated horse or goat anti-mouse antibody was added for 30 min, then incubated with streptavidin (TaKaRa, Dalian, China) for 30 min. Positive staining was detected by Romulin AEC Chromagen (TaKaRa, Dalian, China). To detect the apoptosis rate of hippocampal neurons after exposure to the study drug, terminal deoxynucleotidyl transferase (TdT)-mediated dUTP nick end labeling (TUNEL) staining was performed using a kit in accordance with the manufacturer’s instruction (Applied Biosystems, Foster City, CA, USA). Hippocampal tissues harvested from mice brains were embedded in the OCT compound (Applied Biosystems, Foster City, CA, USA). After snapping refrigeration at − 80 °C, four frozen sections with 8 mm thickness on the cryostat were collected onto one slide. The frozen sections were fixed with 4% paraformaldehyde (Beijing Solarbio Science & Technology Co., Beijing, China) for 15 min. The sections were stained with TUNEL (Beijing Solarbio Science & Technology Co.) in accordance with the manufacturer’s instruction. Biotinylated anti-MCMV early antigen (EA) (Abcam, Cambridge, England, UK) was washed, blocked, and then incubated with the sections at 4 °C overnight. Texas Red-labeled avidin (ThermoFisher, Waltham, MA, USA) was used for binding to the biotin for one hour at 25 °C. These slides were then mounted with the antifade medium containing DAPI (Beijing Solarbio Science & Technology Co.) and observed by a microscope (Wetzlar, Hessen, Germany). Alternatively, the sections were stained with fluorescein isothiocyanate (Beijing Solarbio Science & Technology Co.)-conjugated EA and the subsequent RPE65 antibody provided by Beijing Solarbio Science & Technology Co., or glial fibrillary acidic protein (GFAP) antibody (Beijing Solarbio Science & Technology Co.).

### Cell culture and treatments

The HT22 cell line derived from mouse hippocampal neurons was purchased from Beijing Solarbio Science & Technology Co., Beijing, China. HT22 cells were cultured in DMEM (dulbecco’s modified eagle medium) medium (Sigma-Aldrich, St Louis,MO, USA) supplemented with 10% fetal bovine serum (Sigma-Aldrich). The cell culture plates were incubated at 37 ℃ in a humidified atmosphere containing 5% CO_2_. To develop an *in vitro* propofol injury model as previously described [54], HT22 cells were equally divided into four groups: control group, propofol group (50 µM), propofol (50 µM) + DEX group (50 µM), DEX group (50 µM). In the propofol (50 µM) + DEX (50 µM) group, the HT22 cells was treated with 50 µM DEX, which was followed by administration of 50 µM propofol 30 min later. The HT22 cells were treated with 50 µM propofol for 3 h in both the propofol (50 µM) group and propofol (50 µM) + DEX (50 µM) group. After administration of the study drug, half of the HT22 cells were seeded at 37 ℃ in a humidified atmosphere of 5% CO_2_. HT22 cells were harvested and fixed in cold 80% ethanol after drug treatment, followed by centrifugation and washing, the fixed cells were used for further assays.

### Hoechst 33,258 staining

HT22 cells were seeded onto clean and sterile coverslips placing on 6-well plates, with a density of 2 × 10^5^ cells. After exposure to the indicated drug or the control, the HT22 cells were stained with 0.5 mL Hoechst 33,258 solution (Beyotime, Changsha, China) for 5 min. The morphological changes of HT22 cells involving blue nuclei were observed using fluorescence microscopy (Bioworld Technology, St Louis Park, MN, USA).

### CCK-8 assay and cell growth curves

After corresponding treatments, HT22 cells were suspended until homogeneous distribution, and counted by an automated cell counter (Roche Diagnostics, Mannheim, Germany). 100 μL of cell suspension per well was seeded into a 96-well plateat a density of 3 × 10^4^ cells/mL. Cell viability was detected by the Cell Counting Kit-8 (CCK-8; Sigma-Aldrich) in light of  the manufacturer’s protocol. Briefly, 10 μL of CCK-8 solution was added into each well of the 96-well plate at 0, 6, 12, 24, and 48 h. After two h of culture at 37 ℃ in a humidified atmosphere of 5% CO_2_, optical density (OD) values were detected using a plate reader at 450 nm (Sigma-Aldrich). The cell growth curves were plotted on the basis of the OD values.

### Western blot analysis

Proteins from mouse hippocampi were extracted on ice by lysis buffer (Beyotime, Changsha, China). HT22 cells were lysed by RIPA reagent (Roche Diagnostics, Mannheim, Germany) supplemented with 1 mM phenylmethylsulphonyl fluoride (Sigma-Aldrich, St Louis, MO, USA), and the total protein was obtained after cell lysis. Subsequently, the protein concentration was determined by a BCA (bicinchoninic acid) protein quantification kit (Sigma-Aldrich). Equal quantities of cell lysates were separated by 10% SDS–PAGE (Beyotime) and then transferred onto PVDF membranes (Roche Diagnostics). After blocking with 5% non-fat milk for 1 h at room temperature, the membranes were incubated with primary antibodies at 4 ℃ overnight, which were summarized in Table 1. The membranes were rinsed three times for 10 min each time in 1×tris buffered saline tween (TBST; pH 7.4) at room temperature. Subsequently, the membranes were incubated with horseradish peroxidase-conjugated secondary antibodies (goat anti-rabbit immunoglobulin G; dilution 1:4000; Cell Signaling TechnologyVR, Danvers, MA, USA) for 1 h at room temperature. The membranes were rinsed (3 times/10 min) in 1×TBST (pH 7.4) for chemiluminescence. Images of the immunoblots were acquired by the ChemiDocTM MP Imaging System (Cell Signaling TechnologyVR, Danvers, MA, USA). Experiments were repeated in triplicate.

**Table 1:** Primary antibodies used for western blot analyses

Primary antibodies	MW (kDa)	Dilution	Company	Catalog
DNMT3A	≈102	1:500	Abcam, Shanghai, China	ab228691
DNMT3B	≈95	1:1000	Ptgcn, Chicago, USA	26971-1-AP
Arc	≈45	1:500	Ptgcn, Chicago, USA	16290-1-AP
Caspase-3	≈1735	1:2000	Abcam, Shanghai, China	ab228691
β-actin	42	1:2000	Ptgcn, Chicago, USA	66009-1-Ig

*Abbreviations: MW* molecule weight

### RT-qPCR

Total RNA was extracted from the primary neural stem cells (NSCs) and HT22 cells using TRIZOL reagent (ThermoFisher, Waltham, MA, USA) in accordance with the manufacturer’s instruction. Reverse transcription of mRNA was performed using 1 μg of total RNA, SuperScriptase III (ThermoFisher), and random primers. MiRNA was extracted using RNAiso (TaKaRa, Dalian, China) in light of the manufacturer’s instruction, and reverse transcription of miRNA was performed using the polyadenylated RNA and MirX miRNA First Strand Synthesis kit (Clontech, Nojihigashi, Japan). Expression of mRNA was detected by RT-qPCR using the SYBR Green PCR Kit (Qiagen, Duesseldorf, Germany), and expression of miRNA was determined using the MirX miRNA qRT-PCR SYBR Kit (Clontech). The Stratagene Mx3000P Real-Time PCR System (Agilent, Santa Clara, USA) was used to perform RT-qPCR. The PCR reaction condition was as follows: pre-denaturation at 95 °C for 10 s, followed by 40 cycles of denaturation at 95 °C for 5 s and annealing at 60 °C for 20 s. GAPDH served as the endogenous control for mRNA expression analysis, and U6 was used as the endogenous control in miRNA expression analysis.. The primer sequences in the analyses are shown in Table 2. The relative fold change was calculated using the 2^-ΔΔCt^ method. Each biological sample was tested in triplicate, and all experiments were repeated three times.

**Table 2:** Primer sequences of RT-qPCR

Gene	Sequence
DNMT3A	F: AGAAGCCGCTGTTACCTCTT
DNMT3A	R: GCTGAAACCCTTTGCACAGA
Arc	F: CTGACTCACAACTGCCACAC
Arc	R: TGAGGAAGCCAGATCGTGTT
Caspase-3	F: TCACAGCCGCAACTCAGAC
Caspase-3	G: GGCAGGTCCTGATGAGGTG
β-actin	F: GTGACGTTGACATCCGTAAAGA
β-actin	G: GCCGGACTCATCGTACTCC
miR-377-5p	F: ACACTCCAGCTGGGAGAGGTTGCCCTTGGT
miR-377-5p	G: CTCAACTGGTGTCGTGGAGTCGGCAATTCAGTTGAGGAATTCAC
U6^a^	F: CTCGCTTCGGCAGCACA
U6^a^	G: AACGCTTCACGAATTTGCGT
miR-377-5p^b^	F: AAAATTTTTTTGGGAGAGTTTTTTC
miR-377-5p^b^	G: TTAATAACCATAACCAAACAACGAT
GAPDH^b^	F: CCTTCCCACCCTGTTCATCT
GAPDH^b^	G: AGTTTAGCTGGCCTGGTGAT

*Abbreviations: RT-qPCR* reverse transcription-quantitative polymerase chain reaction, *F* forward primer, *R* reverse primer

^a^ U6, coding gene of U6snRNA

^b^ Primers of promoters

### Dual luciferase reporter gene assay

Bioinformatics analysis by Alggen revealed a putative binding site of miR-377-5p (3’-CTTAAGTGGTTCCCGTTGGAGA-5’) on the *Arc* 3’UTR (the binding sequence is 5’-AGGGCAAC-3’). A wild-type (WT) sequence containing the binding site was synthesized using PCR. Moreover, the potential binding site was mutated in the WT sequence to synthesize a mutant-type (MT) sequence. Both the WT and MT sequences were cloned and then inserted into pGL3 vectors. The vectors were transfected into HT22 cells alone or with Arc over-expression vectors using Lipofectamine 2000 (Qiagen). The HT22 cells were collected at 24 h and the firefly luciferase activity was normalized to renilla luciferase activity.

### DNA Methylation Analysis

DNA methylation analysis was performed through MeDIP assay previously described by Weber, et al. [55]. EpiQuik Hydroxymethylated DNA Immunoprecipitation (hMeDIP) Kit (Epigentek, Wuhan, China) was used for the immunoprecipitation. After interruption with the Covaris sonication system (Covaris, Massachusetts, USA) and subsequent denaturation at 95 ℃ for 10 min, DNA (4 μg) was incubated with the mouse monoclonal antibody (10 μg) against 5-methylcytosine (Ptgcn, Chicago, USA) in 10 × IP buffer (100 mM sodium phosphate with pH 7.0, 1.4 M sodium chloride, 0.5% Triton X-100) at 4 ℃ for 6 h. The complexes of the antibody and DNA were harvested with dynabeads containing anti-mouse IgG (80 μL, Ptgcn) at 4 ℃ for 2 hours on a rotating wheel, and rinsed three times with 1×IP buffer (10 mM sodium phosphate with pH 7.0, 140 mM sodium chloride, 0.05% Triton X-100). The beads were resuspended and incubated in 250 μL of Proteinase K buffer at 50 ℃ for 5 hours, which was composed of 50 mM Tris (pH 8.0), 10 mM EDTA, 0.5% SDS, and 70 μg proteinase K. DNA extracted from the HT22 cells was treated with bisulfite using the EpiTect Bisulfite Kit (Clontech, Nojihigashi, Japan). Then, 20 μL of DNA was used for PCR amplification of the miR-377-5p promoter fragment with primers. The fragment capture was performed using the Methylamp Methylated DNA Capture (MeDIP) Kit (Clontech). After purification, the PCR product was degenerated with the sequencing primers at 80 ℃ for 2 min, followed by pyrosequencing on the PyroMark Q96 instrument (Beijing Solarbio Science & Technology Co.).

For the comparison of DNA methylation of the miR-377-5p promoter region in the DNA fragment isolated from HT22 cells, quantitative real-time PCR was used for verifying the enrichment amount in the promoter region of miR-377-5p for the DNA fragment. Quantitative real-time PCR was performed using the LightCycler 480 Real-Time PCR System, and the reaction mixture consisted of 1× SYBR green master mixture (Roche, Mannheim, Germany), 0.5 mM forward primer, and 0.5 mM reverse primer. A PCR cycle was composed of pre-denaturation at 95 ℃ for 10 min, 45 cycles of denaturation at 95 ℃ for 10 s, followed by annealing at 60 ℃ for 10 s and 72 ℃ for 20 s. Melting curve analysis was performed to quantify the enrichment amount. The methylated-specific primer for the promoter region of miR-377-5p was designed by MethPrimer (http://www.urogene.org/methprimer/). The forward primer sequence was 5’-AAAATTTTTTTGGGAGAGTTTTTTC-3’, and the reverse primer sequence was 5’-TTAATAACCATAACCAAACAACGAT-3’. PCR was performed in accordance with the above-mentioned method.

### Statistical analysis

Data analysis was performed using the SPSS 26.0 software (IBM Corporation, Armonk, NY, USA). Data for the densitometry, TUNEL staining assay, and expression analyses of RNAs and proteins were all expressed as mean ± standard deviation (SD), which reflected the results of independent experiments. The comparison between two groups was performed using Student’s t-test, and the comparisons among multiple groups of duplicate data were performed using one-way ANOVA. In each case the data were evaluated whether they fit the assumption of the test in one-way ANOVA. Bonferroni’s post hoc test was used to perform the comparison between any two means if the *P* value was < 0.05 through one-way ANOVA. *P* values < 0.05 were considered to indicate statistical significance.

## Results

### Bioinformatics analysis for the effects of propofol on the gene expression profiles according to GSE106799 dataset

**Fig. 1:**
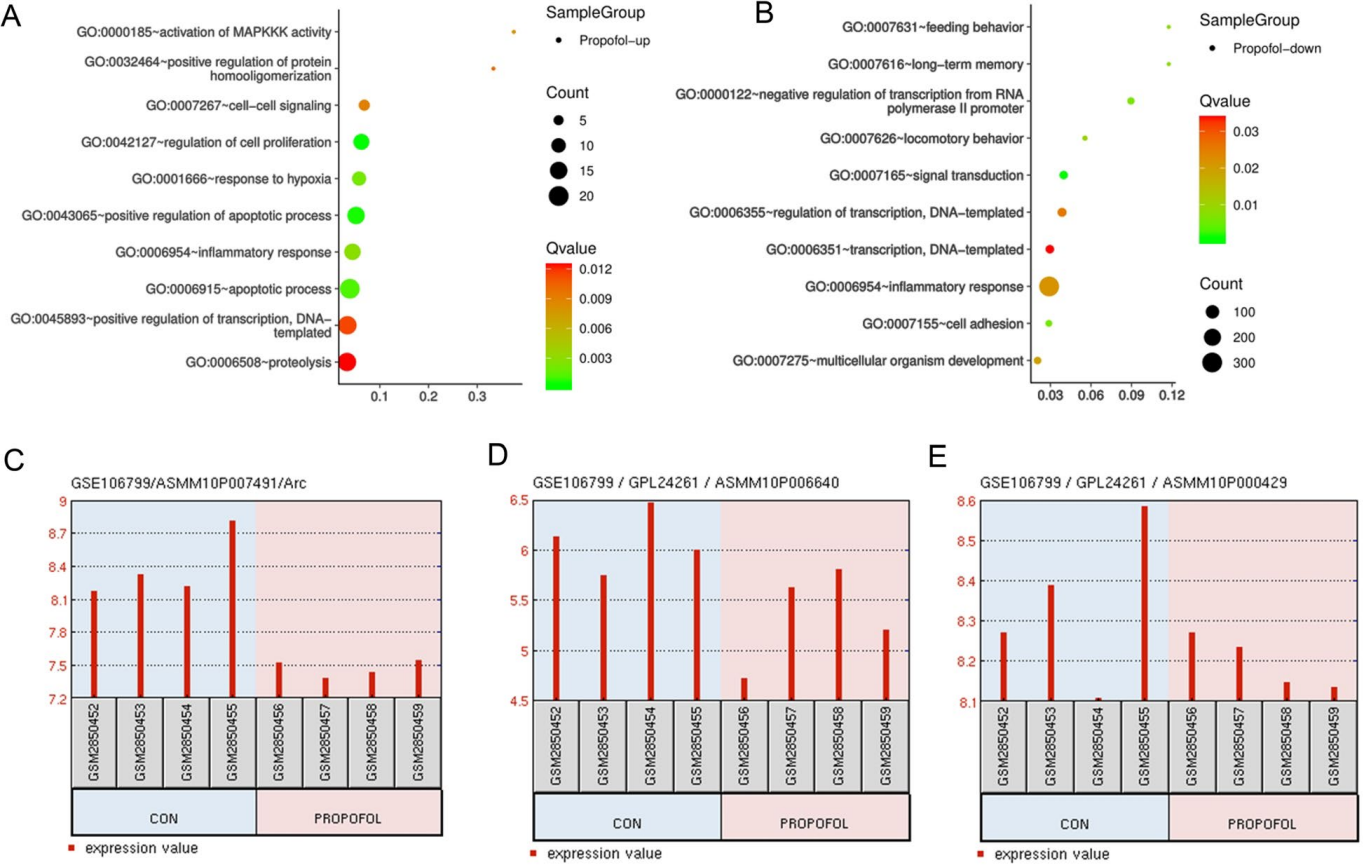
Bioinformatics analysis for effects of propofol on the gene expression profiles according to GSE106799 datasets. Using data from GSE106799 dataset, we performed Gene cluster GO analysis revealed the biological functions of neurons influenced by propofol. **A**, the up-regulated genes after propofol treatment are primarily associated to biological functions, such as cell signal, cell proliferation, response to hypoxia and so on. **B**, the down-regulated genes after propofol treatment are related to biological functions, such as feeding behavior, long-term memory, negative regulation of transcription, and so on. Treatment with propofol decreased the expression of Arc, DNMT3A and DNMT3B (**C**, **D** & **E**, respectively). GO, Gene Ontology

Using data from GSE106799 dataset, we performed gene cluster GO analysis and revealed the biological function how propofol affected neurons. The up-regulated genes after propofol treatment are primarily related to biological functions of cells, such as cell signals, cell proliferation, response to hypoxia, etc. (Fig. 1A). The down-regulated genes following propofol treatment are related to various biological functions, such as feeding behavior, long-term memory, negative regulation of transcription, etc. (Fig. 1B). We found that *Arc* gene located in the following pathways: 0007626~locomotory behavior;0007275~multicellular organism development; 0007616~long-term memory, and Arc was downregulated after propofol treatment. *DNMT3A* and *DNMT3B* located in the pathway: 0000122~negative regulation of transcription from RNA polymerase II promoter. Moreover, propofol treatments decreased the expression of Arc, DNMT3A and DNMT3B (Fig. 1C, D & E).

### DEX protects hippocampal neurons from propofol-induced injury in mice through modulation of the miR-377-5p/Arc pathway

**Fig. 2:**
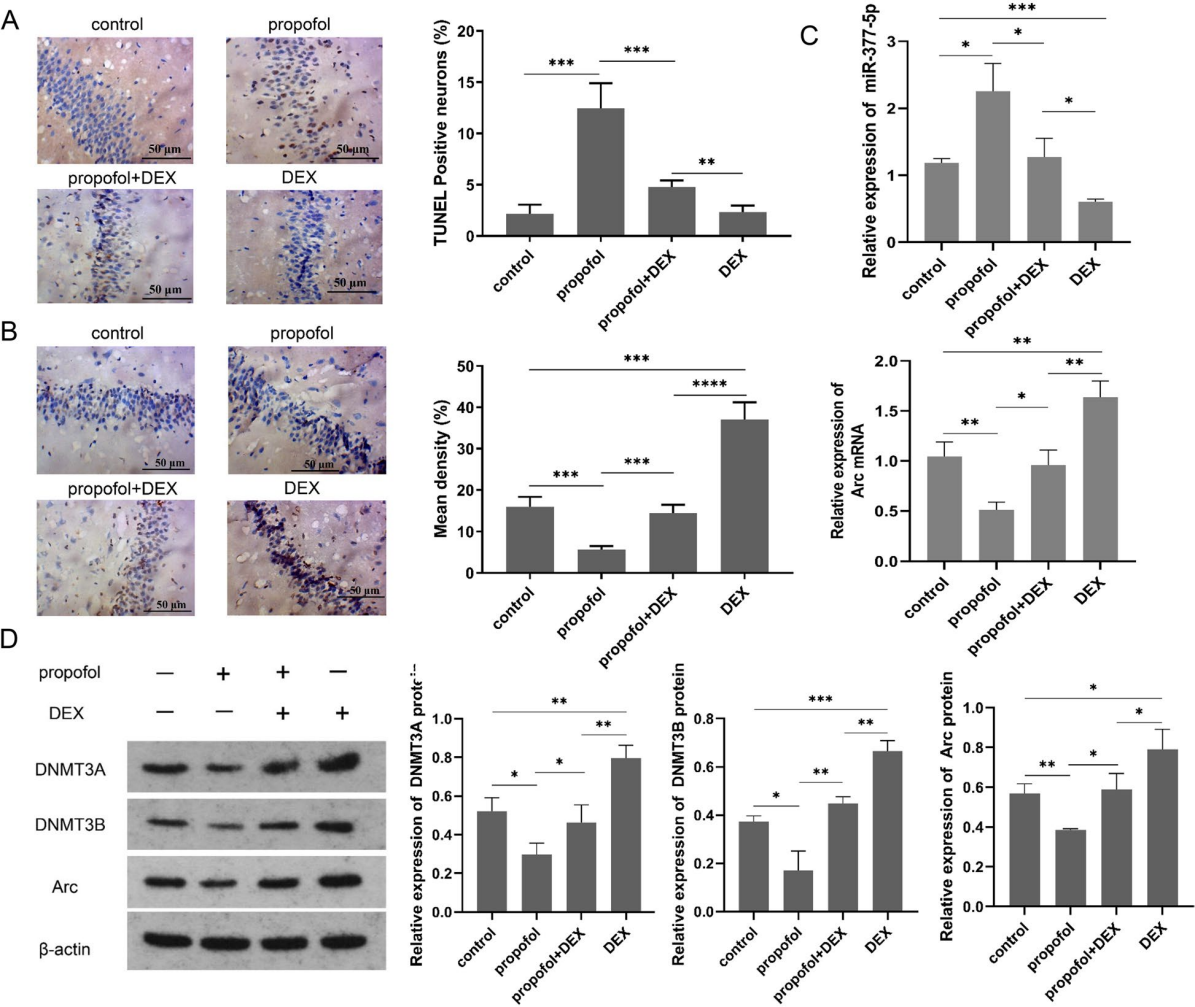
DEX pretreatment reduces the propofol-induced neuronal injury in mouse hippocampus. **A** hippocampal neuronal apoptosis in mouse brains measured with TUNEL staining; **B** Arc positive expression detected with immunohistochemistry in rat hippocampus; **C** miR-377-5p expression and Arc mRNA expression quantified using PR-qPCR; **D** protein levels of DNMT3A, DNMT3B, and Arc measured using western blot analysis, and the full-length blots/gels are presented in Figure S2. Each value represents the mean ± SD for *n* = 3. *, *P* < 0.05; **, *P* < 0.01; ***, *P* < 0.001; ****, *P* < 0.0001. DEX, dexmedetomidine; TUNEL, terminal deoxynucleotidyl transferase (TdT)-mediated dUTP nick end labeling

TUNEL staining was performed to identify apoptotic neurons in the mouse hippocampi (Fig. 2A). Compared with the control group, more TUNEL-stained neurons were observed in the propofol group; TUNEL-stained neurons in the propofol+DEX group were significantly decreased compared with the propofol group. Immunohistochemistry was performed to evaluate Arc positive expression in the mouse hippocampi after drug treatment (Fig. 2B). In addition, Arc mRNA expression was determined using RT-qPCR (Fig. 2C). Compared to the control group, both Arc positive expression and Arc mRNA expression were significantly reduced after propofol treatment, but were elevated following DEX treatment. Moreover, DEX reinstated Arc expression down-regulated by propofol to the normal levels. As indicated by RT-qPCR, miR-377-5p expression was significantly increased after propofol treatment compared to the control group. In contrast, miR-377-5p expression was significantly decreased after DEX treatment compared to the control group. Moreover, miR-377-5p expression in the propofol+DEX group was significantly decreased compared to the propofol group. Western blot analysis showed that propofol significantly decreased DNMT3A, DNMT3B, and Arc protein levels in the mouse hippocampi (Fig. 2D). After DEX treatment, the protein levels of DNMT3A, DNMT3B, and Arc were all significantly increased compared to the control group. There were no significant differences in DNMT3A, DNMT3B, and Arc protein levels between the propofol+DEX group and control group.

### DEX protects HT22 cells against propofol-induced apoptosis via the miR-377-5p/Arc pathway

**Fig. 3:**
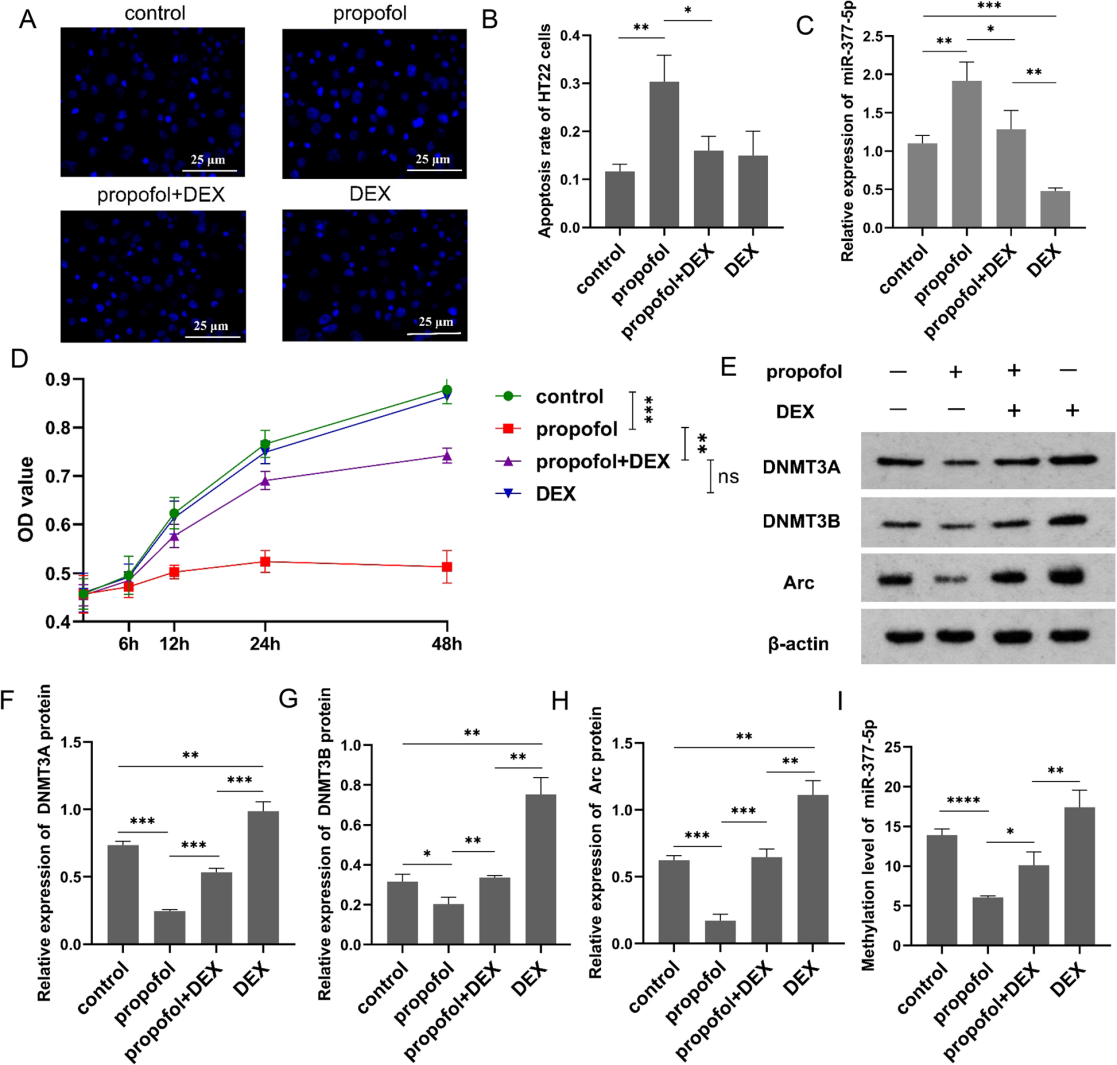
DEX decreases propofol-induced HT22 cell apoptosis via the miR-377-5p/Arc signaling pathway. **A** HT22 cell apoptosis detected using Hochest 33,258 staining; **B** HT22 cell apoptosis rate following exposure to the study drug; **C** miR-377-5p expression levels quantified using PR-PCR; **D** HT22 cell viability and proliferation determined using CCK-8 assay; E, protein levels of DNMT3A (**F**), DNMT3B (**G**), and Arc (**H**) measured using western blot analysis, and the full-length blots/gels are presented in Figure S3. **I** methylation level in the miR-377-5p promoter region detected through MeDIP assay. Each value represents the mean ± SD for *n* = 3. *, *P* < 0.05; **, *P* < 0.01; ***, *P* < 0.001; ****, *P* < 0.0001. DEX, dexmedetomidine; MeDIP, methylated DNA immunoprecipitation

Through Hoechst 33,258 staining, we found cell nucleus shrinkage and chromatin condensation, which exhibited typical apoptotic morphological features after propofol treatment in HT22 cells (Fig. 3A). After exposure to propofol, the percent of apoptotic HT22 cells was significantly increased compared with the control group (Fig. 3B). Whereas compared with the propofol group, the propofol+DEX group showed a significantly decrease in the percent of apoptotic HT22 cells. There were no significant differences in the percent of apoptotic HT22 cells among the control group, propofol+DEX group, and DEX group. RT-qPCR was performed to determine miR-377-5p expression in the HT22 cells after exposure to propofol and/or DEX (Fig. 3C). MiR-377-5p expression in the propofol group was significantly increased compared to the control group, while miR-377-5p expression in the propofol+DEX group was significantly decreased compared to the propofol group. After exposure to DEX, miR-377-5p expression was significantly decreased compared to the control group. HT22 cells were exposed to propofol, propofol+DEX, and DEX, followed by analyses of cell viability at 0, 6, 12, 24, and 48 h using the CCK-8 assay kit (Fig. 3D). After exposure to propofol, HT22 cell viability was significantly inhibited compared to the control group, whereas the introduction of DEX abolished the propofol-induced inhibition. There were no significant differences in the HT22 cell viability among the control group, propofol+DEX group, and DEX group. Western blot analysis was performed to determine the protein levels of DNMT3A, DNMT3B, and Arc in HT22 cells (Fig. 3E). The results showed that exposure to propofol caused significant decreases in the protein levels of DNMT3A (Fig. 3F), DNMT3B (Fig. 3G), and Arc (Fig. 3H), while the introduction of DEX counteracted these decreases. The protein levels of DNMT3A, DNMT3B, and Arc in the propofol+DEX group were all significantly increased compared to the propofol group. After exposure to DEX, the protein levels of DNMT3A, DNMT3B, and Arc were all significantly increased compared to the control group. DNA methylation in the miR-377-5p promoter was analyzed through MeDIP assay (Fig. 3I). The methylation level in the miR-377-5p promoter was significantly decreased after exposure to propofol, compared to the control group. While compared to the propofol group, the methylation level in the miR-377-5p promoter was significantly elevated in the propofol+DEX group. The methylation level was significantly increased in the DEX group compared to the control group.

### Arc expression is regulated by the DNMT3A/miR-377-5p pathway

**Fig. 4:**
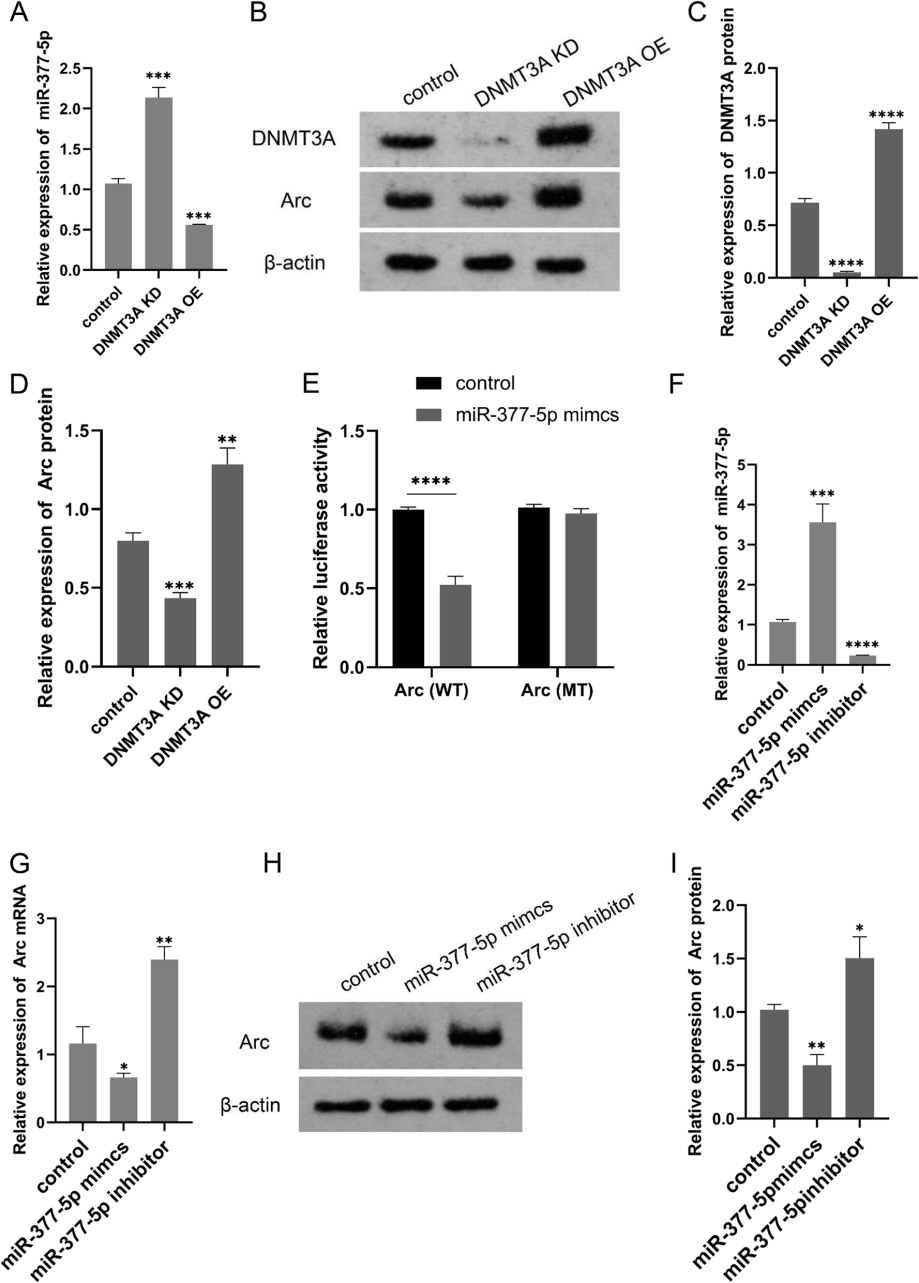
Arc is regulated by the DNMT3A/miR-377-5p pathway. **A** Arc mRNA expression after DNMT3A knockdown or overexpression quantified using RT-PCR. **B** Arc protein expression after DNMT3A knockdown or overexpression identified using western blot analysis, and the full-length blots/gels are presented in Figure S4. **C** the changes of DNMT3A protein expression after DNMT3A knockdown or overexpression; **D** Arc protein expression quantified using western blot analysis. **E** targeted regulation of miR-377-5p on Arc identified through luciferase reporter assay. **F** miR-377-5p expression levels after the introduction of miR-377-5p mimcs or inhibitor quantified using PR-PCR; **G** Arc mRNA expression levels quantified using RT-PCR; **H** the changes of Arc protein expression after the introduction of miR-377-5p mimcs or inhibitor identified using western blot analysis, and the full-length blots/gels are presented in Figure S5; **I** Arc protein levels detected using western blot analysis. Each value represents the mean ± SD for *n* = 3. *, *P* < 0.05; **, *P* < 0.01; ***, *P* < 0.001; ****, *P* < 0.0001. DEX, dexmedetomidine; KD, knockdown; OE, overexpression; WT, wild-type; MT, mutant-type

As indicated by RT-qPCR, miR-377-5p expression was significantly increased in the HT22 cells with DNMT3A knockdown but decreased in the HT22 cells with DNMT3A overexpression (Fig. 4A). Western blot analysis showed that Arc protein levels were significantly decreased and increased in the HT22 cells with DNMT3A knockdown and DNMT3A overexpression, respectively (Fig. 4B, C, and D). To investigate the effect of miR-377-5p on Arc expression, we performed the dual luciferase reporter gene assay to determine the interaction between miR-377-5p and Arc 3’UTR. HT22 cells were co-transfected with miR-377-5p mimics and luciferase reporter constructs containing WT or MT Arc 3’UTR, and the relative luciferase activity was measured and normalized to that of the negative control cells (Fig. 4E). After transfection of miR-377-5p mimics, the luciferase activity of Arc-WT construct declined to 47%, while the luciferase activity of Arc-MT construct was not affected. RT-qPCR assays were performed to determine the expression levels of miR-377-5p and Arc after transfection of miR-377-5p mimics and inhibitors. MiR-377-5p mimics caused a significant increase in miR-377-5p expression (Fig. 4F), and a significant decrease in Arc expression compared to the control group (Fig. 4G); After transfection with miR-377-5p inhibitors, miR-377-5p expression was significantly reduced while Arc expression was significantly elevated. Western blot analysis (Fig. 4H) showed that Arc protein levels were decreased and increased after transfection with miR-377-5p mimics and inhibitors, respectively (Fig. 4I).

### DEX attenuates propofol-induced HT22 cell apoptosis by targeting Arc

**Fig. 5:**
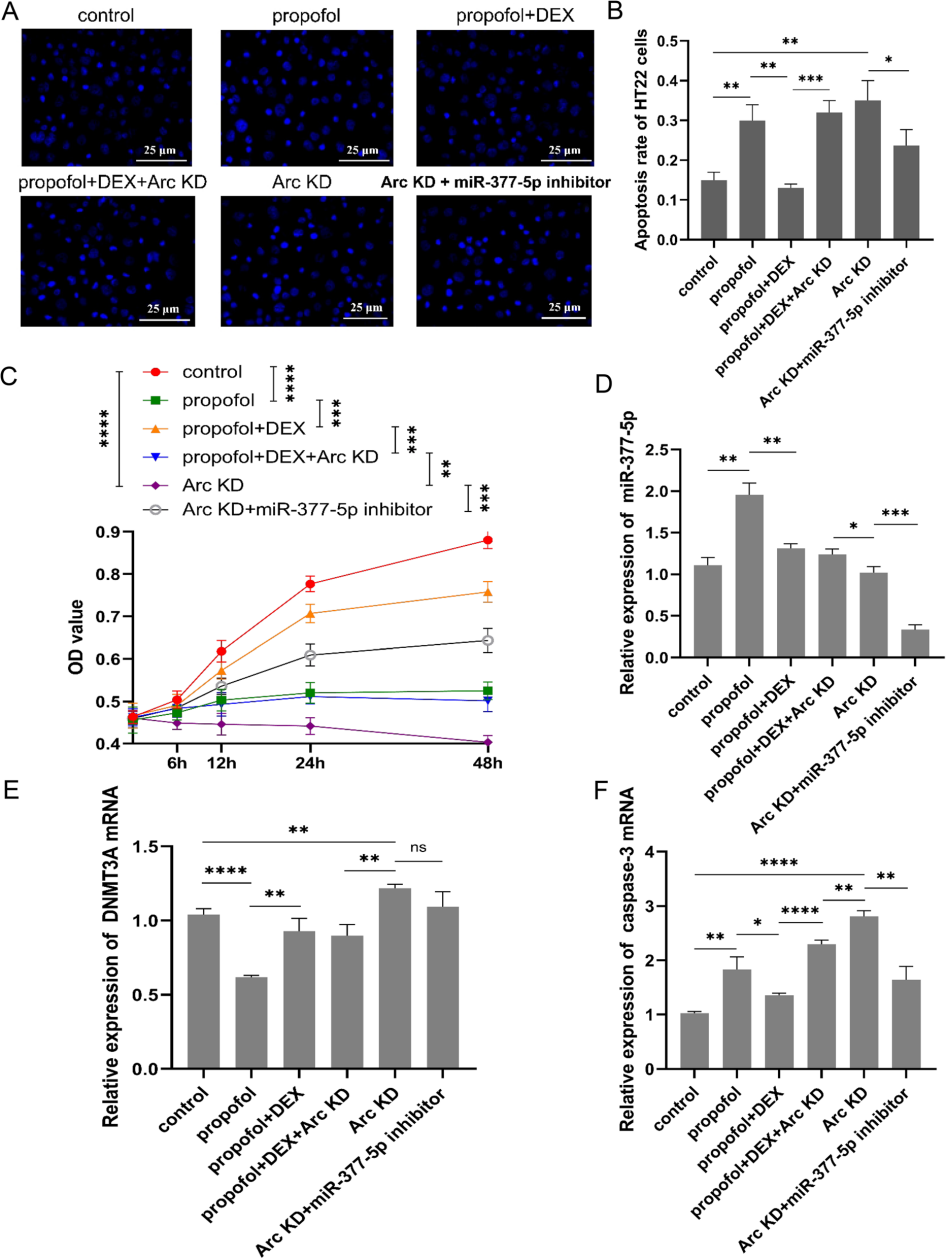
DEX protects HT22 cells against propofol-induced apoptosis by regulating Arc expression. **A** HT22 cell apoptosis detected using Hochest 33,258 staining; **B** HT22 cell apoptosis rate following exposure to the study drug or shArc; **C** HT22 cell viability and proliferation determined through CCK-8 assay; the mRNA expression levels of miR-377-5p (**D**), DNMT3A (**E**), and caspase-3 (**F**) quantified using RT-qPCR. Each value represents the mean ± SD for *n* = 3. *, *P* < 0.05; **, *P* < 0.01; ***, *P* < 0.001; ****, *P* < 0.0001. DEX, dexmedetomidine; KD, knockdown

**Fig. 6:**
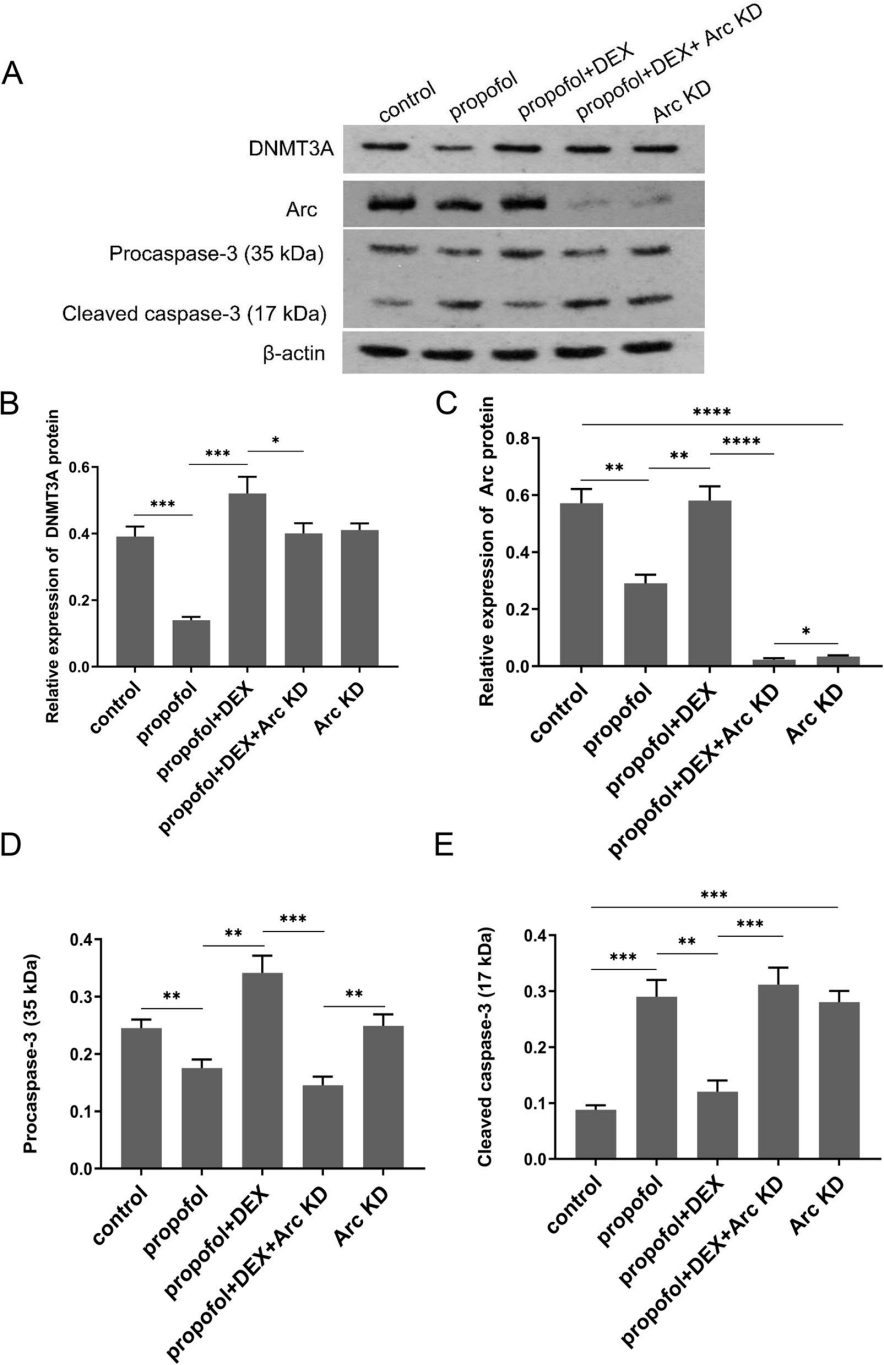
Arc is associated with apoptosis-related protein expression. **A** protein expression levels of DNMT3A (**B**), Arc (**C**), caspase-3-35 (**D**), and caspase-3-17 (**E**) detected using western blot analysis, and the full-length blots/gels are presented in Figure S6. Furthermore, the full-length membranes with membrane edges visible for all protein expression tests and the other two replicate experiments in the western blot analysis are shown in Figure S7 and Figure S8, respectively. Each value represents the mean ± SD for *n* = 3. *, *P* < 0.05; **, *P* < 0.01; ***, *P* < 0.001; ****, *P* < 0.0001. DEX, dexmedetomidine; KD, knockdown

In order to verify that the protective effect of DEX on neurons against propofol-induced apoptosis was related to Arc, we performed Arc knockdown alone or in combination with DEX plus propofol treatment. In addition, Arc knockdown combined with transfection of miR-377-5p inhibitors was performed. Hochest 33,258 staining assays were performed to detect the HT22 cell apoptosis after indicated treatments (Fig. 5A). DEX suppressed propofol-induced apoptosis of HT22 cells, however the anti-apoptotic effect of DEX was diminished after Arc knockdown. Silencing Arc independently elevated the percentage of apoptotic cells as well. Moreover, transfection of miR-377-5p inhibitors reduced the apoptosis of HT22 cells caused by Arc knockdown (Fig. 5B). CCK-8 assay was performed to analyze the HT22 cell viability and proliferation (Fig. 5C). DEX improved the HT22 cell viability that was suppressed by propofol. However, this protective effect of DEX was also abolished by Arc knockdown. Without exposure to the study drugs, depletion of Arc also decreased the HT22 cell viability. After transfection with miR-377-5p inhibitors, the HT22 cell viability was improved compared to Arc knockdown alone. RT-qPCR analysis showed that propofol decreased DNMT3A expression but increased miR-377-5p expression, however these effects of propofol were reversed by DEX independent of Arc knockdown or not. Moreover, simultaneous intervention of miR-377-5p and Arc significantly decreased miR-377-5p expression compared to Arc knockdown alone as well as the other groups, but had no significant effect on the expression of DNMT3A (Fig. 5D and E). Depletion of Arc diminished the effect of DEX decreasing propofol-induced up-regulation of caspase-3. Transfection of miR-377-5p inhibitors lessened the up-regulation of caspase-3 caused by Arc knockdown alone (Fig. 5F). As indicated by western blot assay, propofol decreased DNMT3A and Arc protein levels, however these effects of propofol were reversed by DEX (Fig. 6A, B and C). DNMT3A protein level was not affected by Arc knockdown. Propofol induced reduction of procaspase-3 but augmentation of cleaved caspase-3. DEX reversed these actions of propofol, but this effect of DEX was not observed after Arc knockdown (Fig. 6D and E).

## Discussion

Propofol is widely used for general anesthesia especially in intensive care settings [56]. However, propofol-induced cognitive impairment is a prevalent clinical concern [57, 58]. In a rat model, propofol exerted neurotoxicity to the developing brain, because it induced hippocampal neuron apoptosis that contributed to the cognitive impairment [59]. Berndt N, et al. [60] found that propofol suppressed complex II of the respiratory chain in the CA3 hippocampal area of rats. In the present study, propofol induced hippocampal neuron apoptosis both *in vitro* and *in vivo*, and significant decreases in the expression levels of Arc, DNMT3A, and DNMT3B.

DEX has been identified to attenuate propofol-induced neurotoxicity to hippocampal neurons derived from rats via several signaling pathways, such as Erk1/2/CREB/BDNF, PI3k/Akt/GSK3β, GSK-3β/CRMP2, CDK5/CRMP2, and miR-34a/SIRT1/PI3K/Akt signaling pathways, and the previous studies indicated that propofol induced hippocampal neuron injury by elevating apoptosis-related protein expression [54, 61–66]. As a protective mechanism, DEX reduced propofol-induced hippocampal neuron injury in rat brains by reducing miR-34a expression and then improving SIRT1 expression, resulting in activation of the PI3K/Akt pathway [54].

In the present study, we illustrated that propofol treatment caused decreases in DNMT3A and DNMT3B expression, which lowered the methylation level in the miR-377-5p promoter. As a result, miR-377-5p expression was increased, leading to the deficiency of Arc that was the target of miR-377-5p. However, DEX treatment enhanced the expression of DNMT3A and DNMT3B, elevated the methylation level in the miR-377-5p promoter, and decreased miR-377-5p expression, leading to augment of Arc expression. Eventually, the introduction of DEX attenuated propofol-induced hippocampal neuron injury.

This study found that DNMT3A and DNMT3B played crucial roles in propofol-induced neurotoxicity as well as DEX-mediated neuroprotection. Actually, DNA methyltransferases (DNMTs) catalyze DNA methylation and modulate gene expression in the central nervous system [67]. DNMT inhibitors have been reported to have a potential effect on learning involving inhibition of maintenance of long-term potentiation (LTP) [68–70]. Changes in the expression of DNMT3A and DNMT3B are correlated with cognitive rehabilitation as well as neuroprotection in AD [71]. In mature neurons, DNMTs expression was maintained at a high level, and DNMT3A knockdown induced the synaptic alteration and learning deficit, which directly influenced learning and memory behavior. Thus, DNMT3A in the postmitotic neuron is a key regulator in memory formation [72]. DNMT3A loss causes widespread transcriptional alterations and severe impairment of neuronal functions [73], and DNMT3A haploinsufficiency in the brain leads to neurodevelopmental disorders involved in growth and behavioral alterations [74]. Recently, it has been revealed that hypoxic preconditioning exerted anti-hypoxic neuroprotection and maintained HT22 cell proliferation and viability through downregulation of the expression of DNMT3A and DNMT3B [75]. Whereas our study illustrated that high expression of DNMT3A was associated with DEX-mediated neuroprotection against propofol-induced hippocampal neuron injury. These results seemed contradictory, maybe due to an essential difference between drug treatment and hypoxic preconditioning.

MiR-377-5p was first found by Lucherini OM, et al. [76] that miR-377-5p expression was related to serum amyloid A circulating levels. Afterwards, it was reported that miR-377-5p expression might be implicated in the pathogenesis of latent tuberculosis infection and the recurrence score of breast carcinomas with positive estrogen receptor [77, 78]. Recently, miR-377-5p overexpression was found to inhibit cell development (viability, proliferation, metastasis, and invasion) and regulate cell cycle distribution in lung cancer [79, 80]. Moreover, miR-377-5p overexpression aggrandized myocardial dysfunction as well as apoptosis, and promoted the release of inflammatory factors [81]. However, miR-377-5p downregulation suppressed the proliferation and invasion of HepG2 cells belonging to hepatocellular carcinoma cell lines [82]. Interestingly, Li Y, et al. [83] has demonstrated that miR-377-5p was up-regulated after propofol treatment and contributed to induce neurotoxicity, the result was consistent with our finding.

## Conclusions

This study indicated that propofol induced hippocampal neuron injury characterized by hippocampal neuronal apoptosis and decreased neuronal viability. DEX protected hippocampal neuron against propofol-induced injury by restoring the expression levels of DNMT3A, miR-377-5p, and Arc to the normal levels. Potentially, our findings contribute to provide novel ideas in the development of new drugs for attenuating or eliminating clinical adverse reactions caused by propofol-induced neurotoxicity to hippocampal neurons.

## Supplementary Information

**Additional file 1.**

**Additional file 2.**

**Additional file 3.**

**Additional file 4.**

**Additional file 5.**

**Additional file 6.**

**Additional file 7.**

**Additional file 8.**

## Data Availability

The datasets used and/or analyzed during the current study are available from the corresponding author on reasonable request.

## Abbreviations

DEX: Dexmedetomidine
TUNEL: Terminal deoxynucleotidyl transferase (TdT)-mediated dUTP nick end labeling
CCK-8: Cell Counting Kit-8
MeDIP: Methylated DNA immunoprecipitation
BDNF: Brain derived neurotrophic factor
NMDA: N-methyl d-aspartate
PPP3CA: Protein phosphatase 3 catalytic subunit alpha
METTL3: Methyltransferase like 3
CREB: CAMP responsive element binding
Arc: Activity-regulated cytoskeletal
Egr1: Early growth response 1
IEGs: Immediate early genes
AD: Alzheimer disease
DNMT3A: DNA methyltransferase 3 alpha
EA: Early antigen (EA)
GFAP: Glial fibrillary acidic protein
DMEM: Dulbecco's modified eagle medium
OD: Optical density
BCA: Bicinchoninic acid
SDS–PAGE: Sodium dodecyl sulphate–polyacrylamide gel electrophoresis
PVDF: Polyvinylidene difluoride
NSC: Neural stem cell
WT: Wild-type
MT: Mutant-type
SD: Standard deviation
DNMTs: DNA methyltransferases
LTP: Long-term potentiation
MW: Molecule weight
RT-qPCR: Reverse transcription-quantitative polymerase chain reaction
U6: Coding gene of U6snRNA
GO: Gene Ontology
