# Supplementary material for: Dexmedetomidine reduces propofol-induced hippocampal neuron injury by modulating the miR-377-5p/Arc pathway
Source: BMC Pharmacol Toxicol. 2022 Mar 25;23:18. doi: 10.1186/s40360-022-00555-9 (PMC8957152; doi:10.1186/s40360-022-00555-9)

Figure S2. Full-length blots/gels for protein expression detection of DNMT3A, DNMT3B, and Arc after drug treatment in mouse hippocampus.

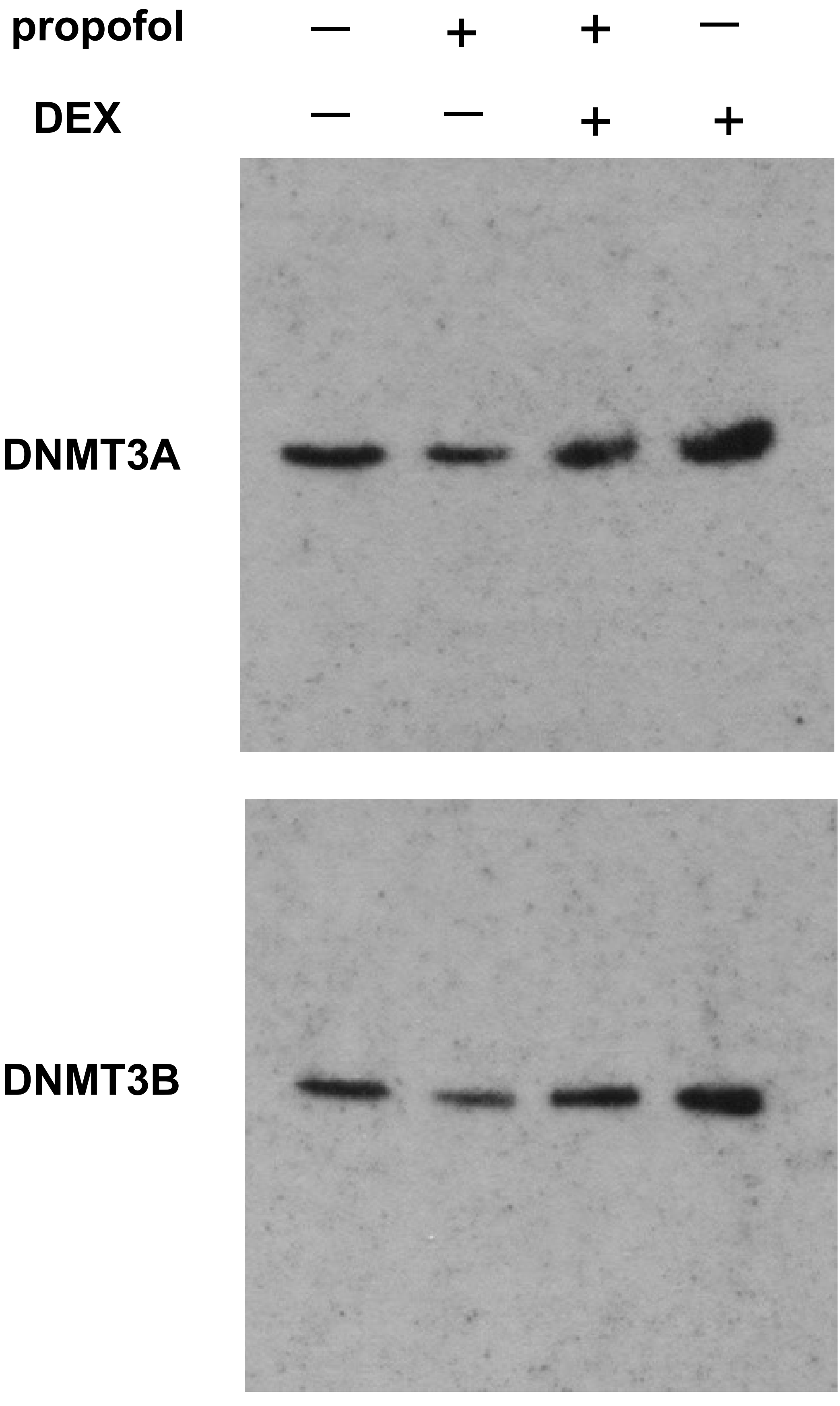

Arc

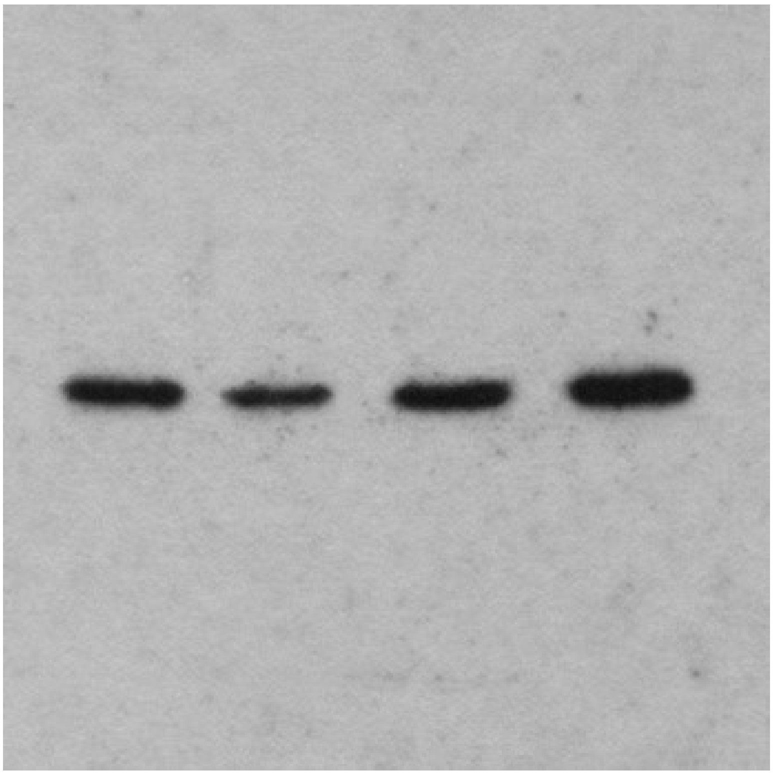

β-actin

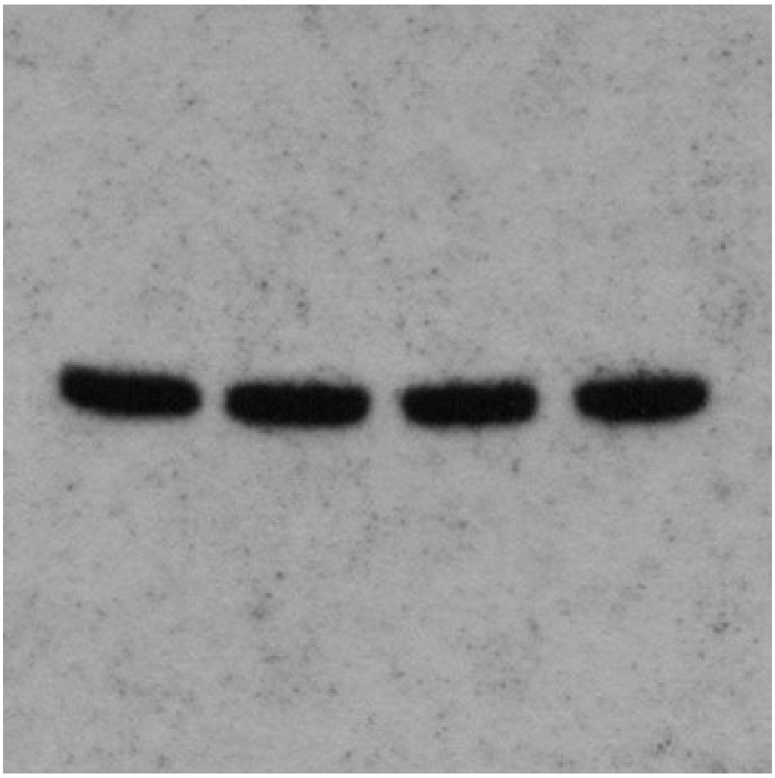

Supplement: Supplementary file 2 — Additional file 2. [file 40360_2022_555_MOESM2_ESM.pdf]
