# Supplementary material for: Dexmedetomidine reduces propofol-induced hippocampal neuron injury by modulating the miR-377-5p/Arc pathway
Source: BMC Pharmacol Toxicol. 2022 Mar 25;23:18. doi: 10.1186/s40360-022-00555-9 (PMC8957152; doi:10.1186/s40360-022-00555-9)

Figure S3. Full-length blots/gels for protein expression determination of DNMT3A, DNMT3B, and Arc following exposure to the study drug in HT22 cells.

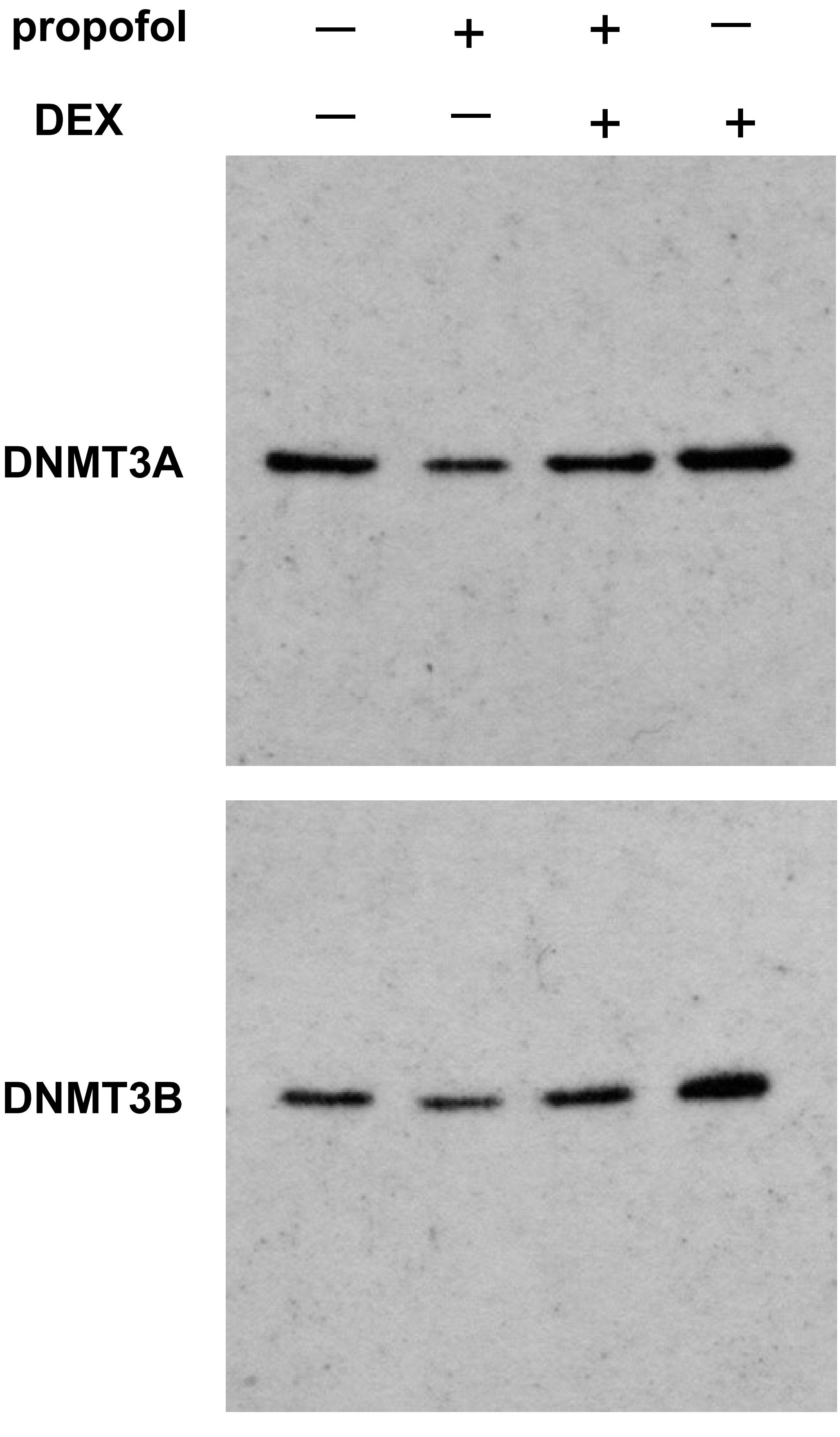

Arc

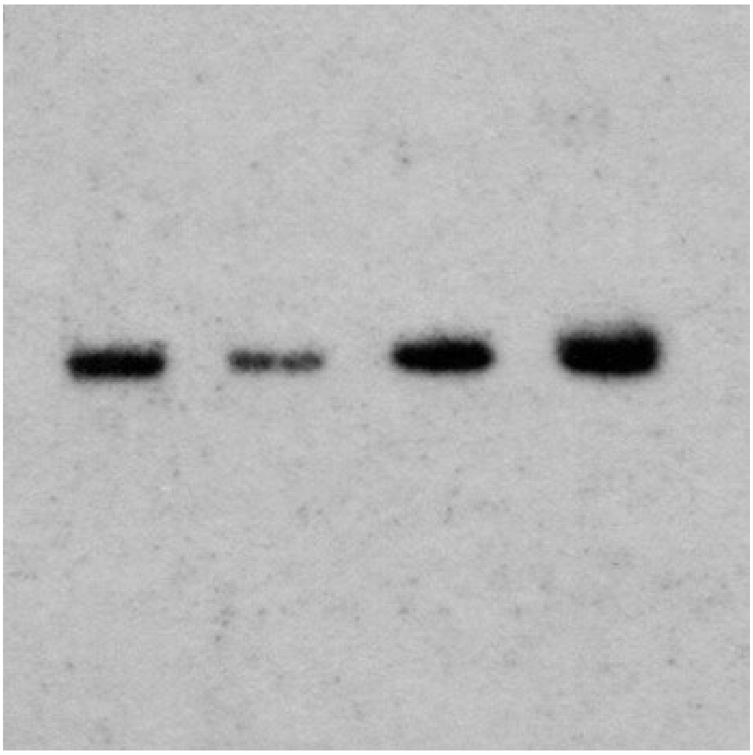

β-actin

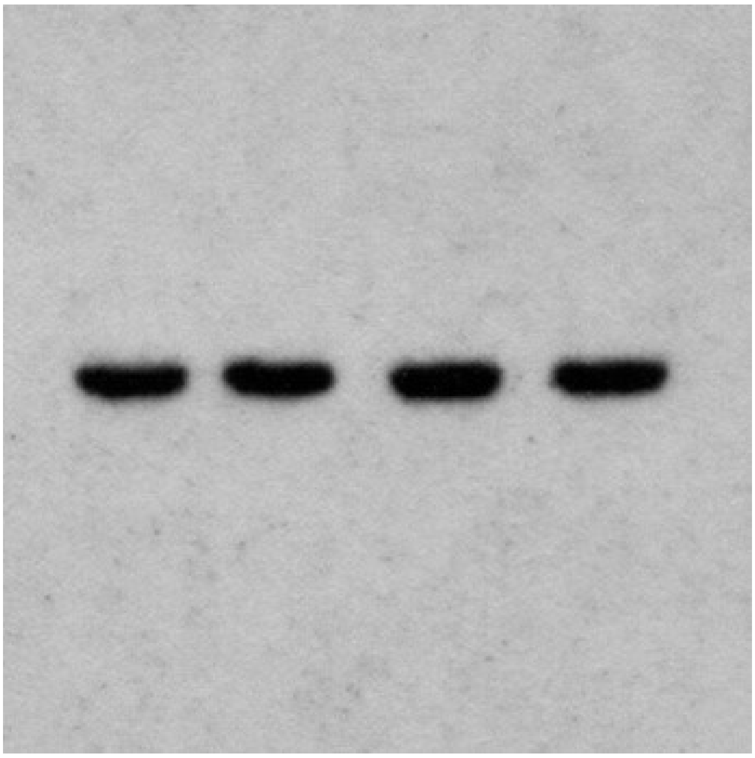

Supplement: Supplementary file 3 — Additional file 3. [file 40360_2022_555_MOESM3_ESM.pdf]
