# Supplementary material for: Dexmedetomidine reduces propofol-induced hippocampal neuron injury by modulating the miR-377-5p/Arc pathway
Source: BMC Pharmacol Toxicol. 2022 Mar 25;23:18. doi: 10.1186/s40360-022-00555-9 (PMC8957152; doi:10.1186/s40360-022-00555-9)

Figure S4. Full-length blots/gels for protein expression analysis of Arc after DNMT3A knockdown and overexpression in HT22 cells.

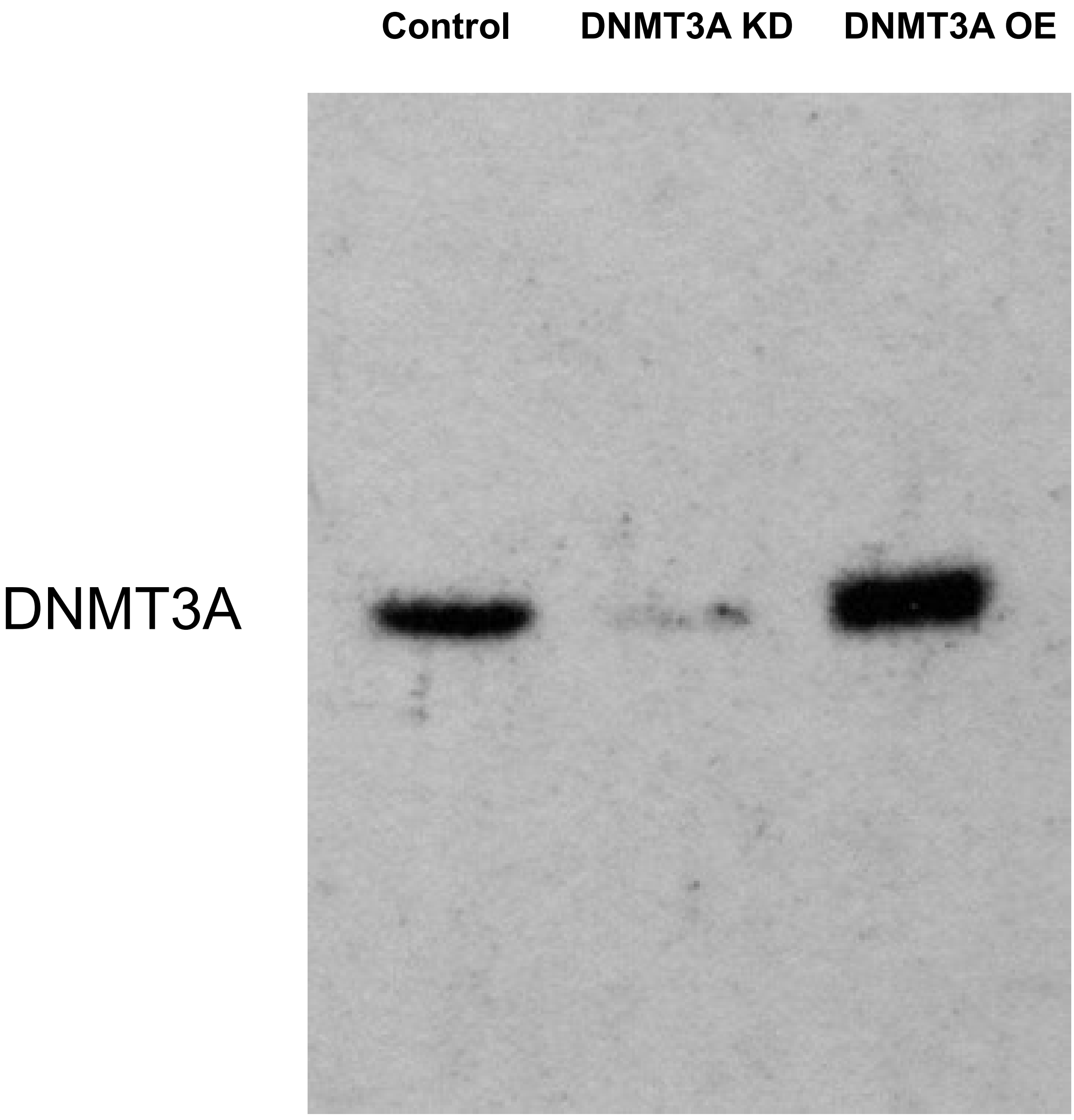

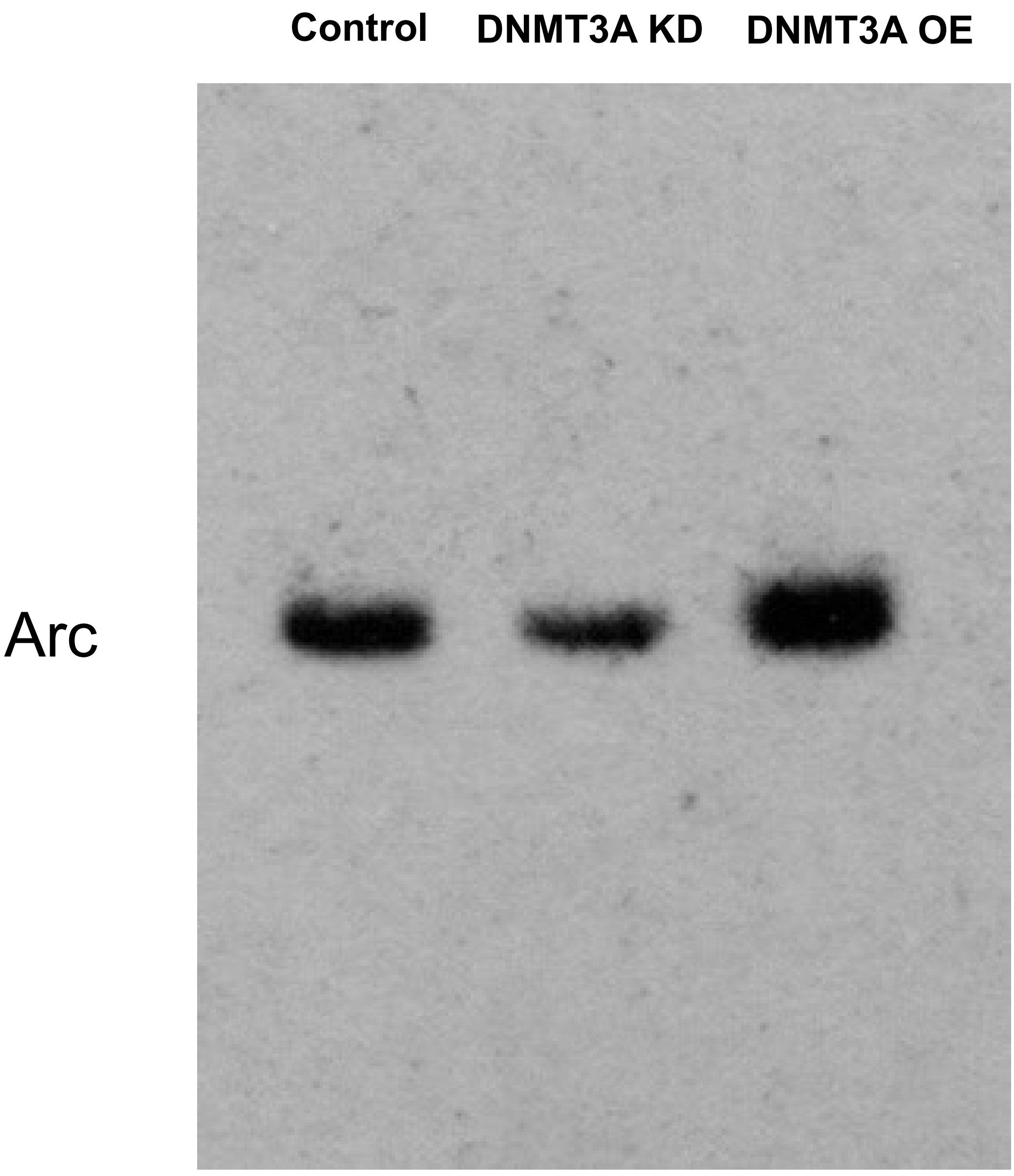
Control
DNMT3A KD
DNMT3A OE
Arc

Control DNMT3A KD DNMT3A OE

β-actin

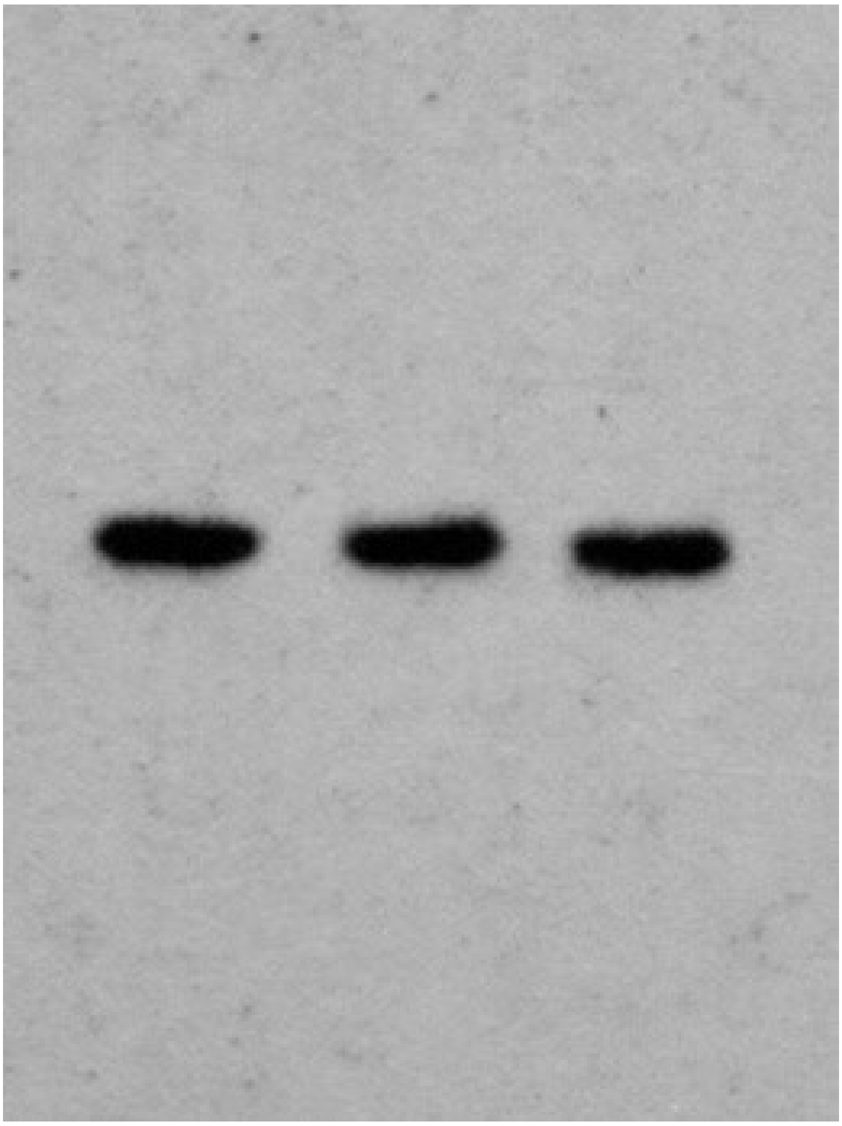

Supplement: Supplementary file 4 — Additional file 4. [file 40360_2022_555_MOESM4_ESM.pdf]
