# Supplementary material for: Dexmedetomidine reduces propofol-induced hippocampal neuron injury by modulating the miR-377-5p/Arc pathway
Source: BMC Pharmacol Toxicol. 2022 Mar 25;23:18. doi: 10.1186/s40360-022-00555-9 (PMC8957152; doi:10.1186/s40360-022-00555-9)
Supplement: Supplementary file 5 — Additional file 5. [file 40360_2022_555_MOESM5_ESM.pdf]

Figure S5. Full-length blots/gels for protein expression analysis of Arc after introduction of miR-377-5p mimcs or inhibitor in HT22 cells.

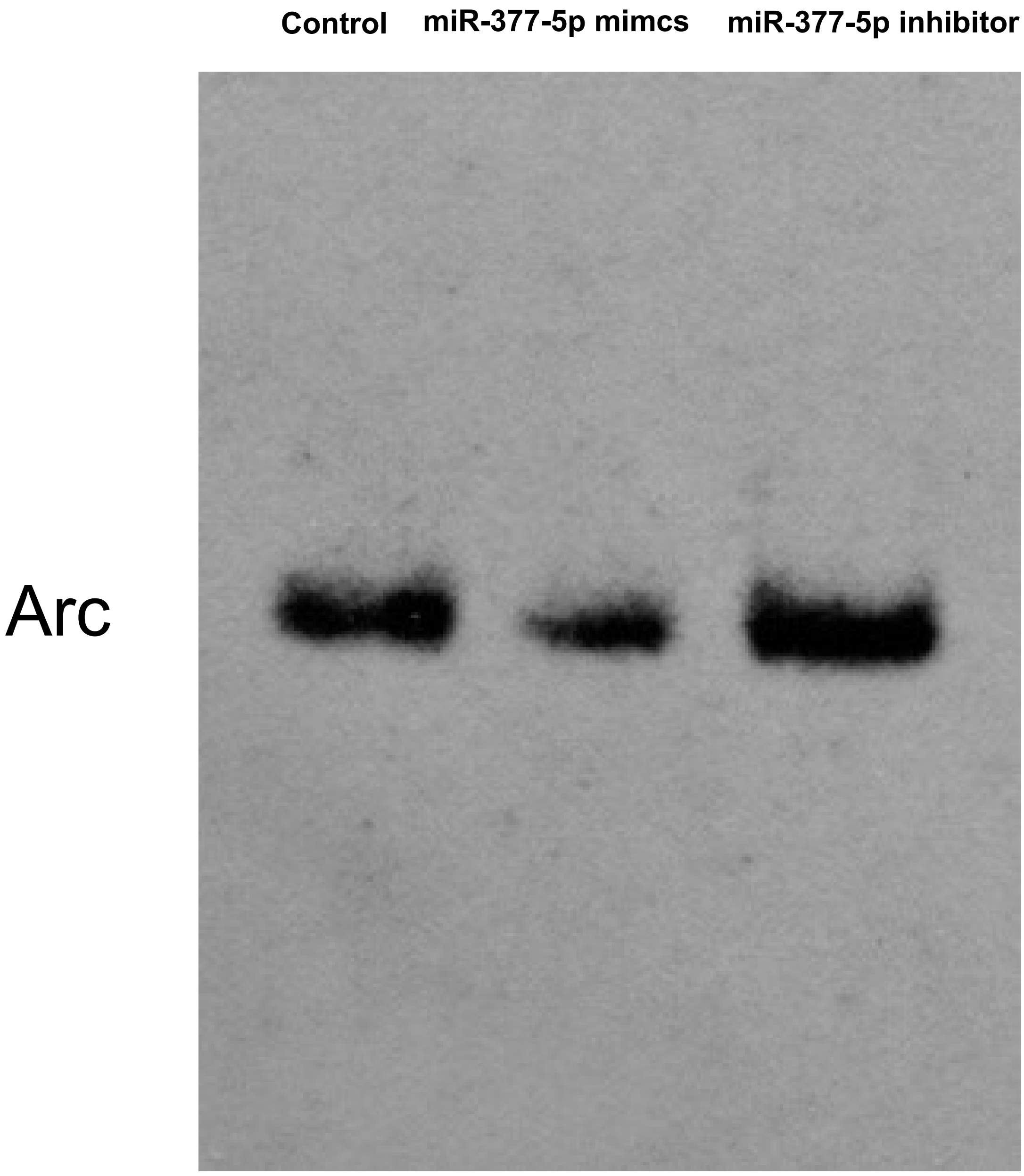

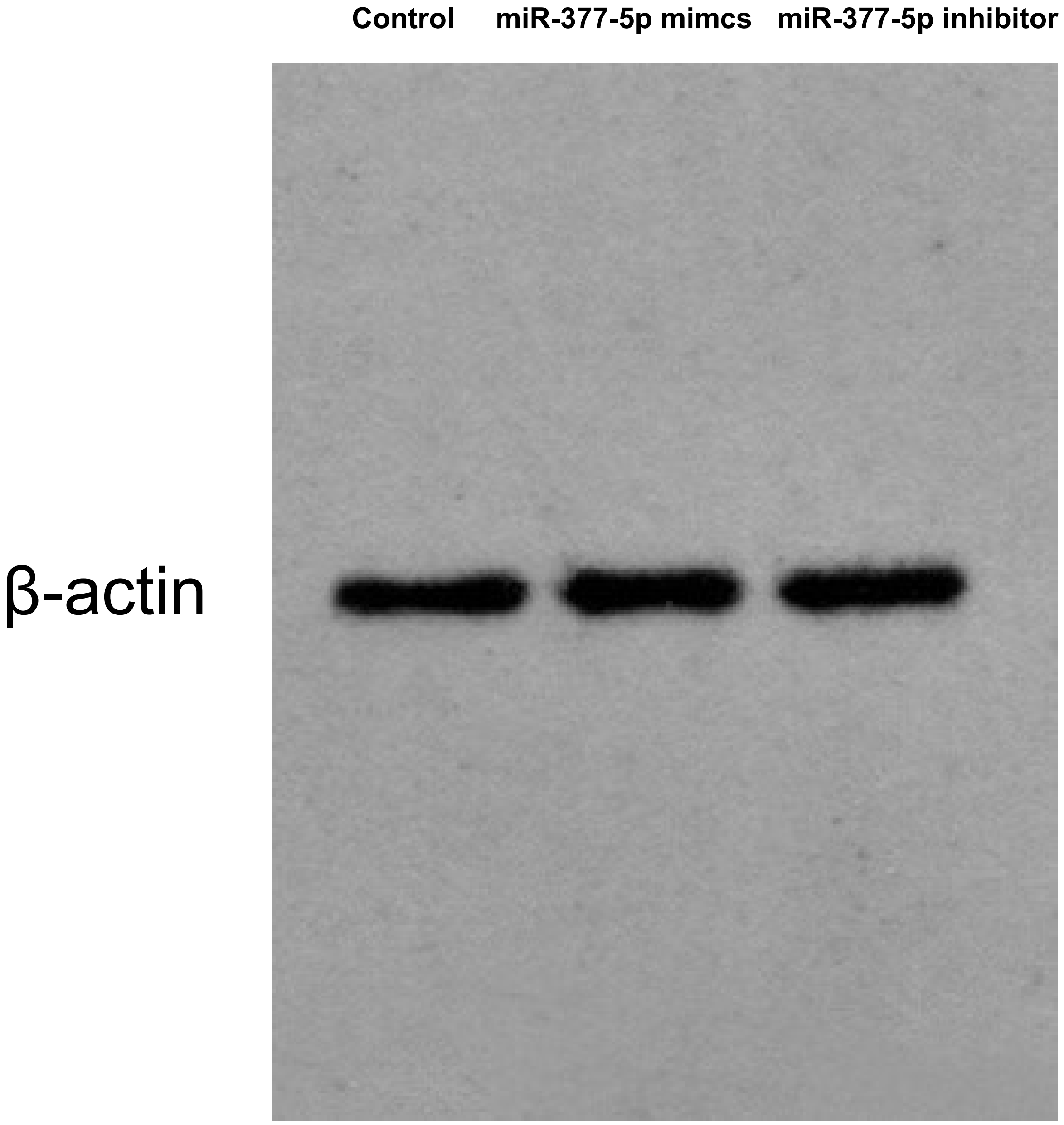
Control
miR-377-5p mimcs
miR-377-5p inhibitor
β-actin
