# Supplementary material for: Dexmedetomidine reduces propofol-induced hippocampal neuron injury by modulating the miR-377-5p/Arc pathway
Source: BMC Pharmacol Toxicol. 2022 Mar 25;23:18. doi: 10.1186/s40360-022-00555-9 (PMC8957152; doi:10.1186/s40360-022-00555-9)
Supplement: Supplementary file 6 — Additional file 6. [file 40360_2022_555_MOESM6_ESM.pdf]

Figure S6. Full-length blots/gels for protein expression tests of DNMT3A, Arc, caspase-3-35, and caspase-3-17 after drug treatment and/or Arc knockdown in HT22 cells .

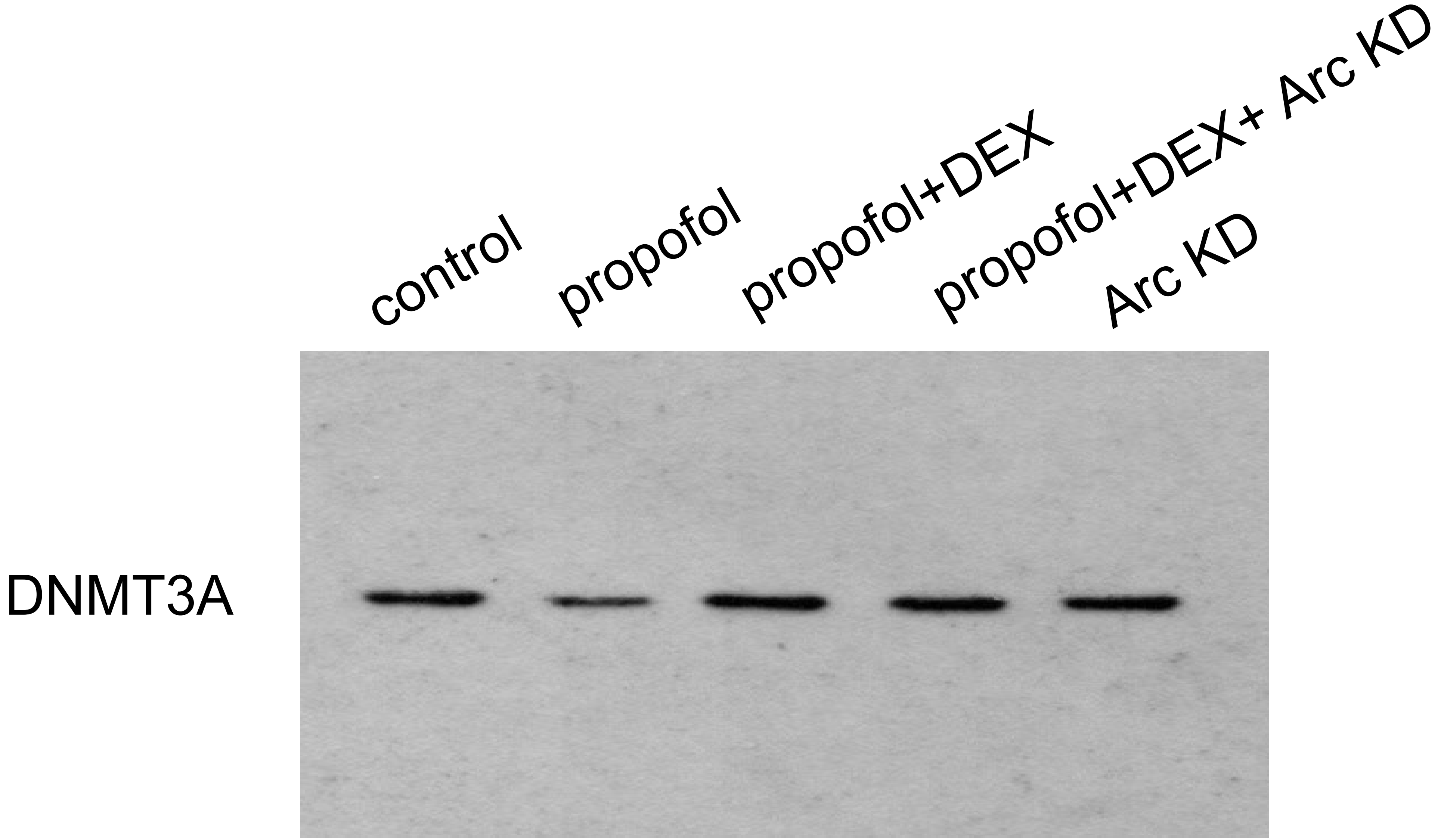
control
propofol
propofol+DEX
propofol+DEX+ Arc KD
Arc KD
DNMT3A

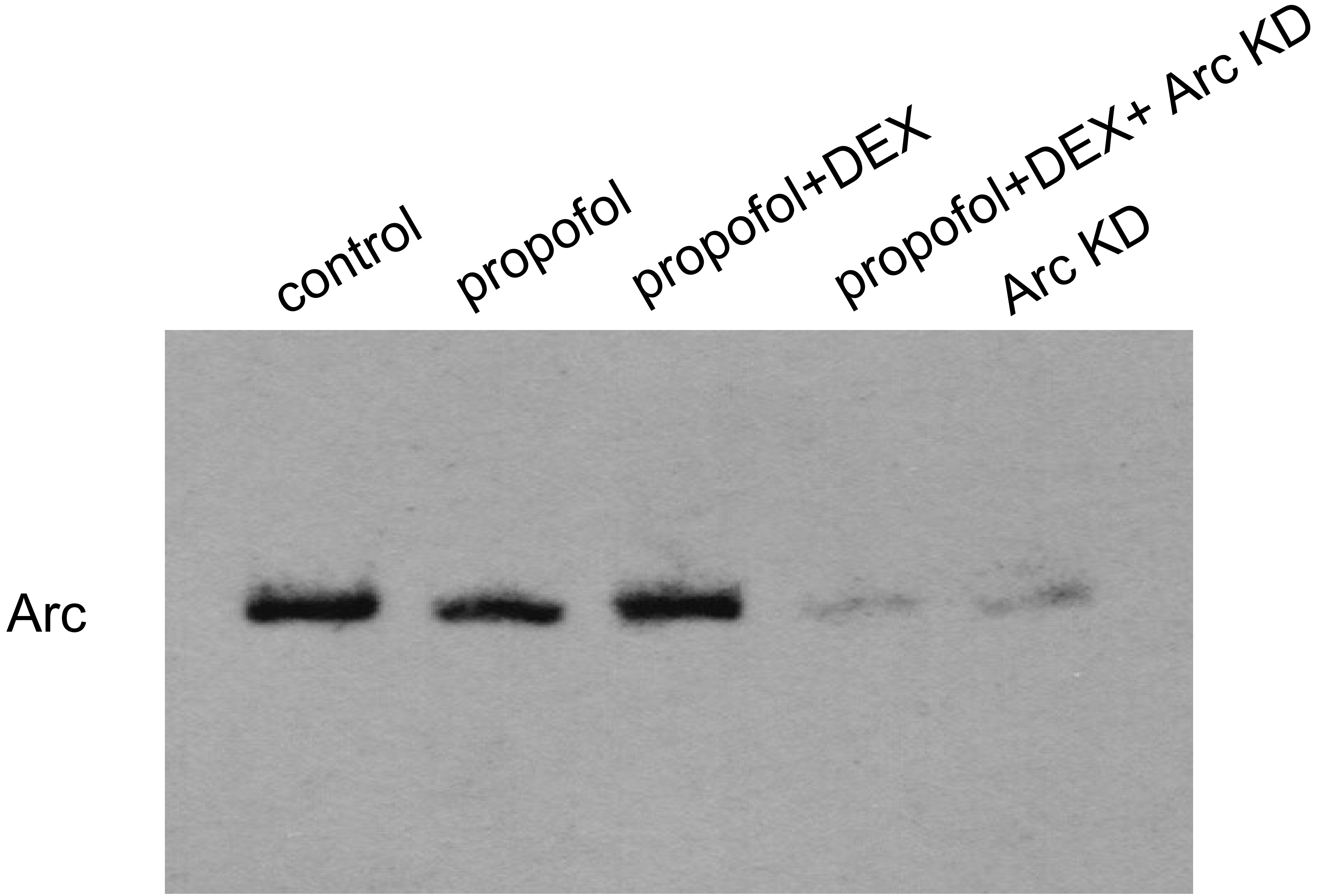
control
propofol
propofol+DEX
propofol+DEX+ Arc KD
Arc KD
Arc

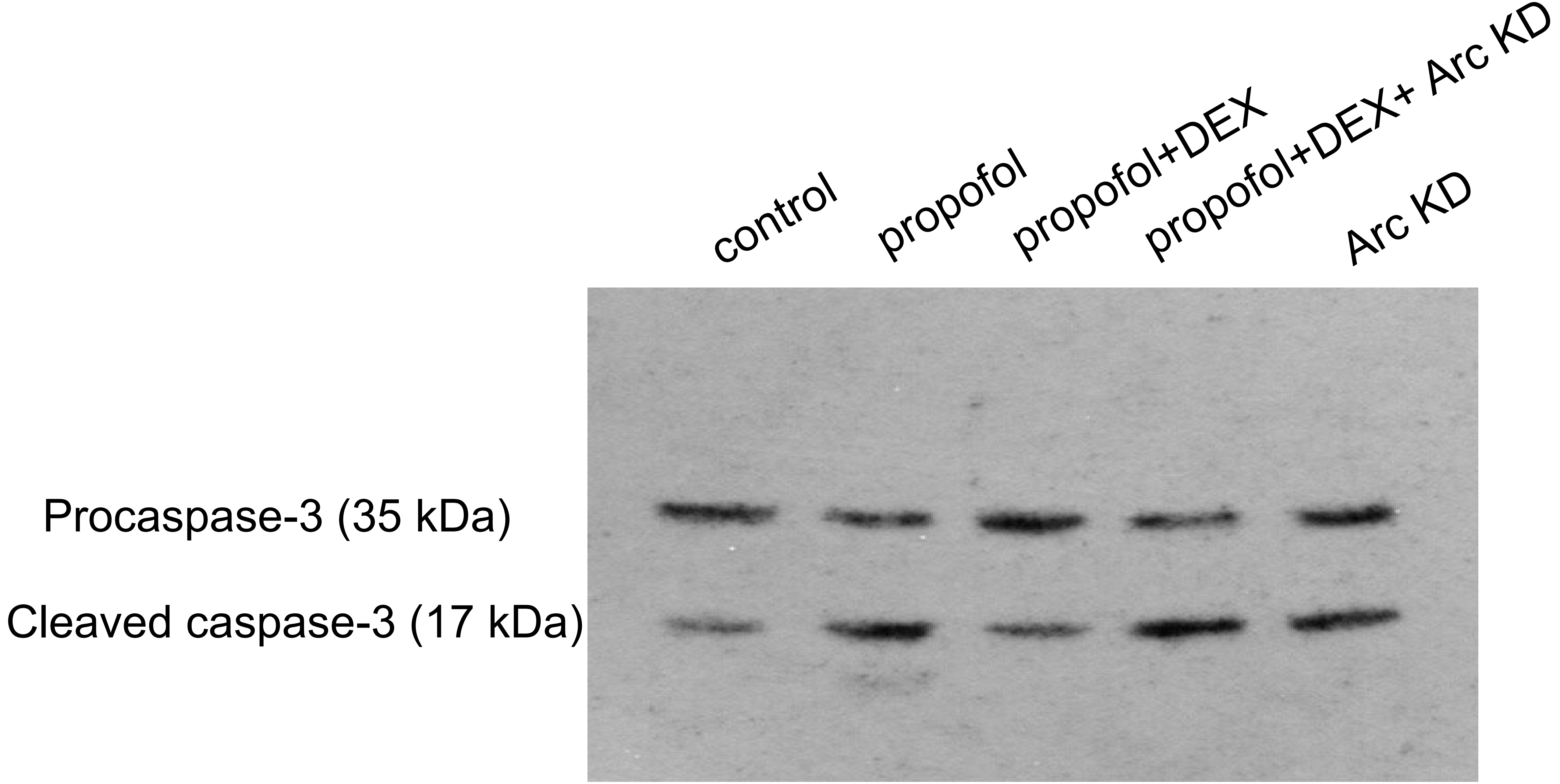

control
propofol
propofol+DEX
propofol+DEX+ Arc KD
Arc KD
Procaspase-3 (35 kDa)
Cleaved caspase-3 (17 kDa)

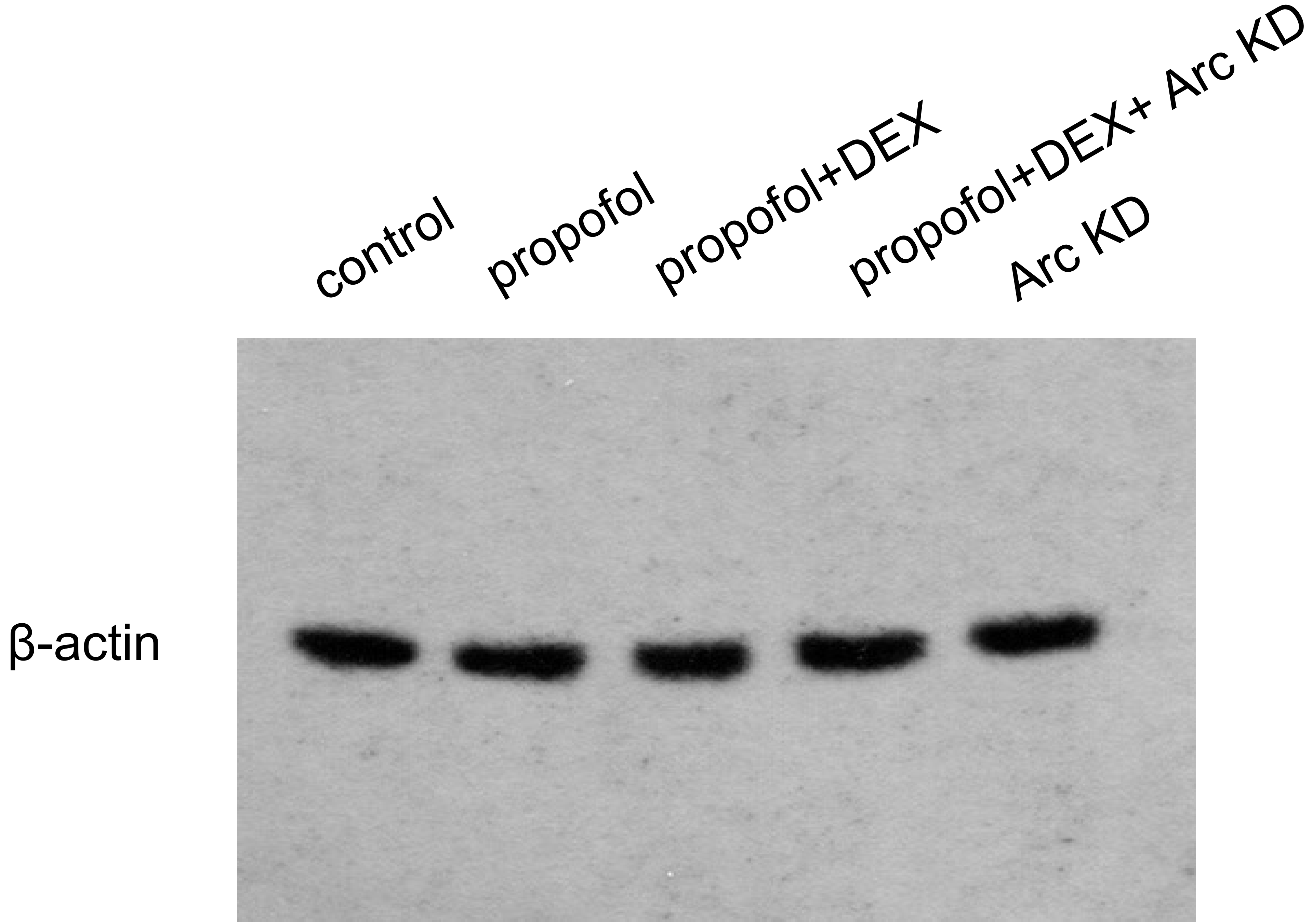
control
propofol
propofol+DEX
propofol+DEX+ Arc KD
Arc KD
β-actin
