# Supplementary material for: Dexmedetomidine reduces propofol-induced hippocampal neuron injury by modulating the miR-377-5p/Arc pathway
Source: BMC Pharmacol Toxicol. 2022 Mar 25;23:18. doi: 10.1186/s40360-022-00555-9 (PMC8957152; doi:10.1186/s40360-022-00555-9)

Figure S7. Full-length membranes with membrane edges visible for all protein expression tests.

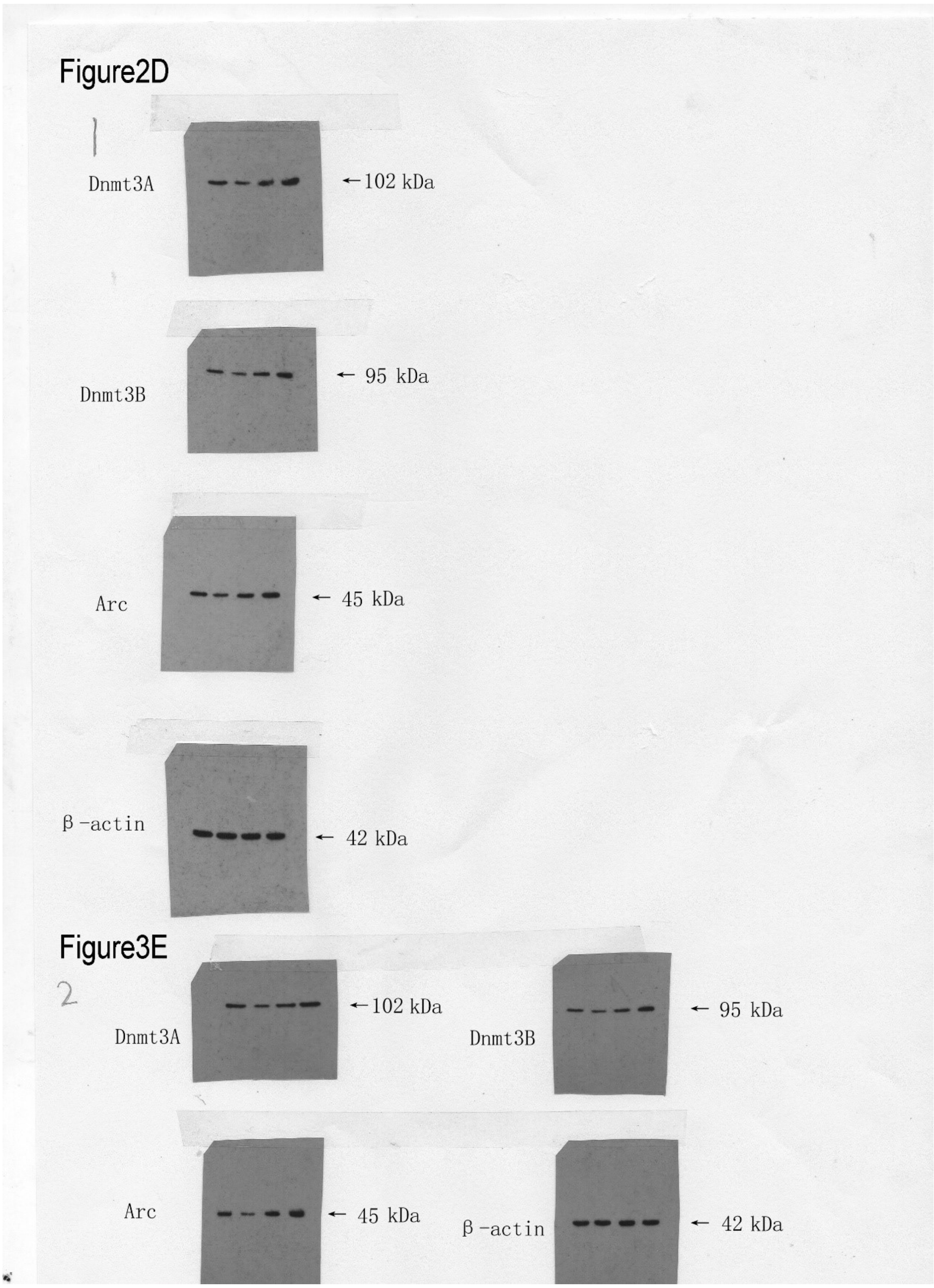

Figure4B

3

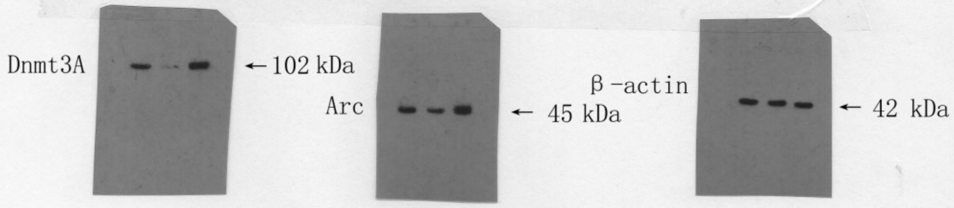

4

Figure4H

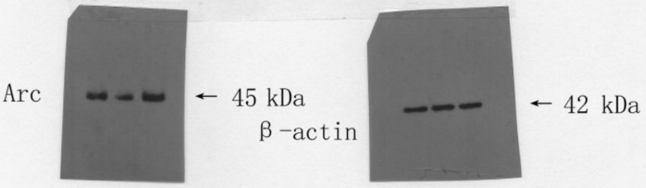

Figure6A

5

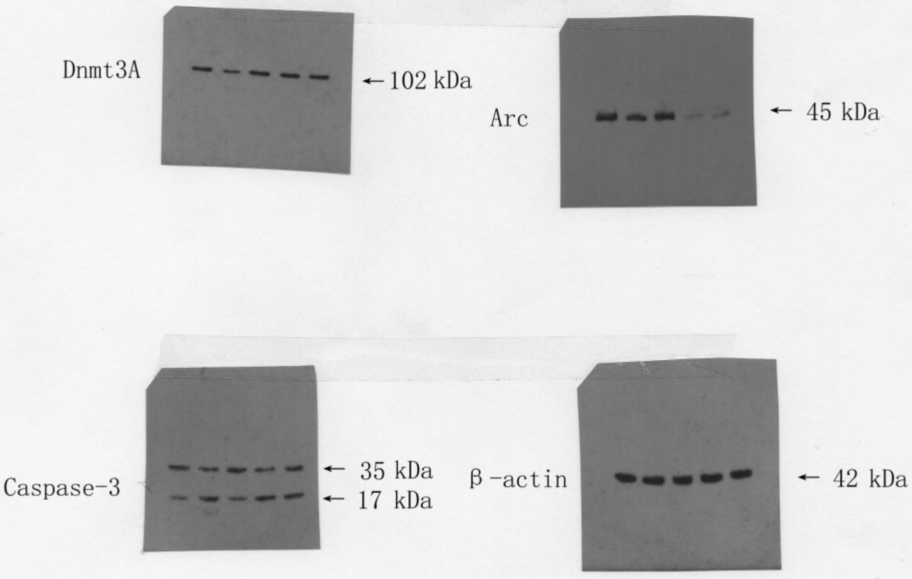

Supplement: Supplementary file 7 — Additional file 7. [file 40360_2022_555_MOESM7_ESM.pdf]
