# Supplementary material for: Dexmedetomidine reduces propofol-induced hippocampal neuron injury by modulating the miR-377-5p/Arc pathway
Source: BMC Pharmacol Toxicol. 2022 Mar 25;23:18. doi: 10.1186/s40360-022-00555-9 (PMC8957152; doi:10.1186/s40360-022-00555-9)
Supplement: Supplementary file 8 — Additional file 8. [file 40360_2022_555_MOESM8_ESM.zip › 1-Figure S8.pdf]

Figure S8. Full-length membranes with membrane edges visible for the other two replicate experiments in western blot analysis

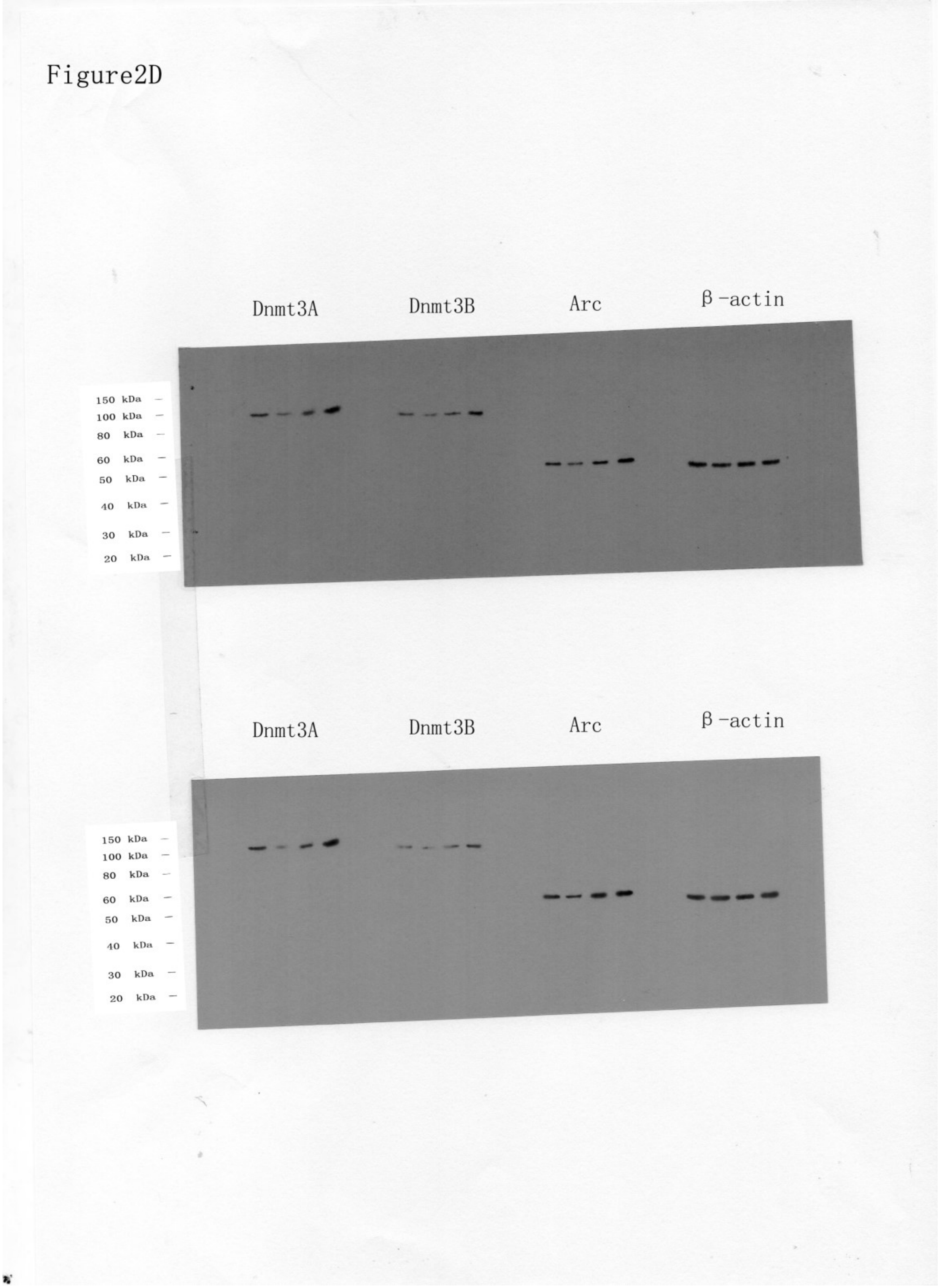

Figure3E

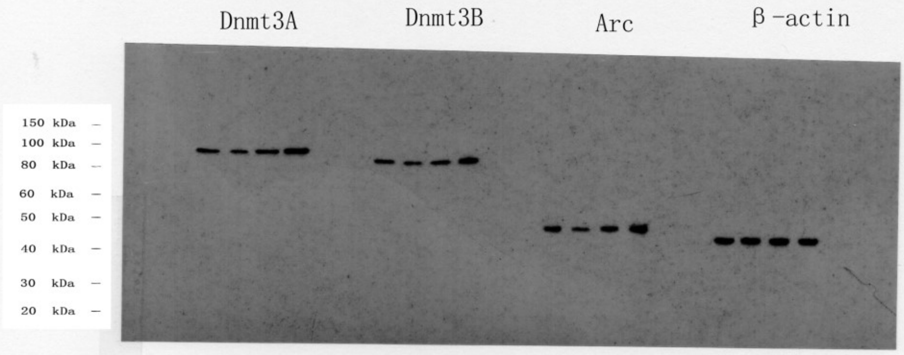
Dnmt3A
Dnmt3B
Arc
β-actin
150 kDa
100 kDa
80 kDa
60 kDa
50 kDa
40 kDa
30 kDa
20 kDa

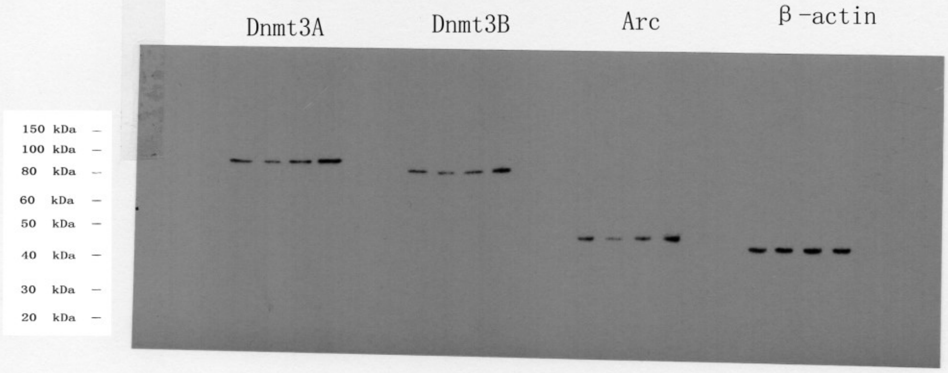
Dnmt3A
Dnmt3B
Arc
β-actin
150 kDa
100 kDa
80 kDa
60 kDa
50 kDa
40 kDa
30 kDa
20 kDa

Figure4B

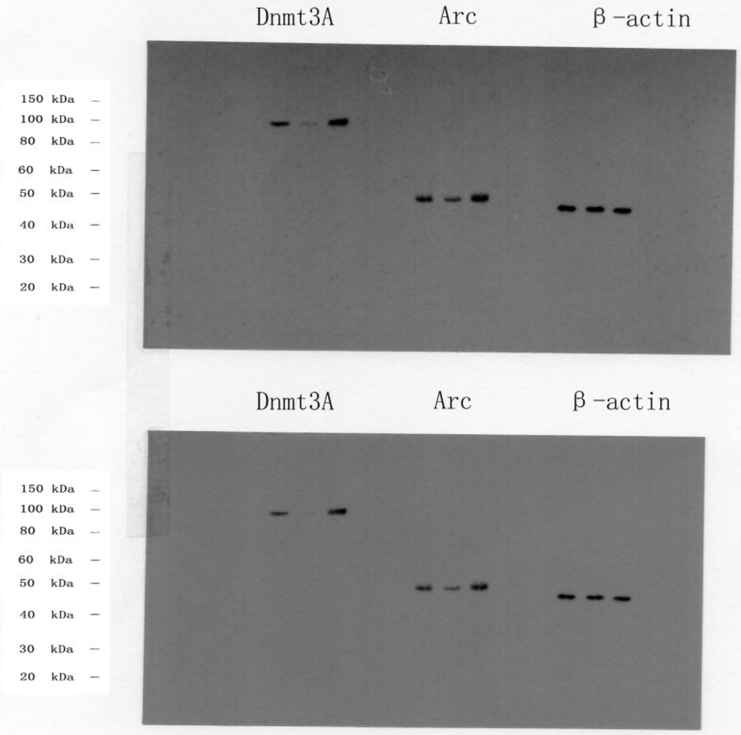

Figure4H

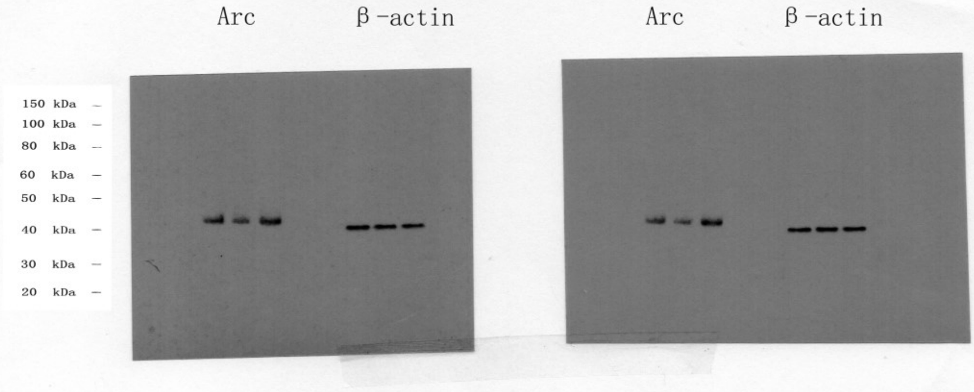

Figure6A

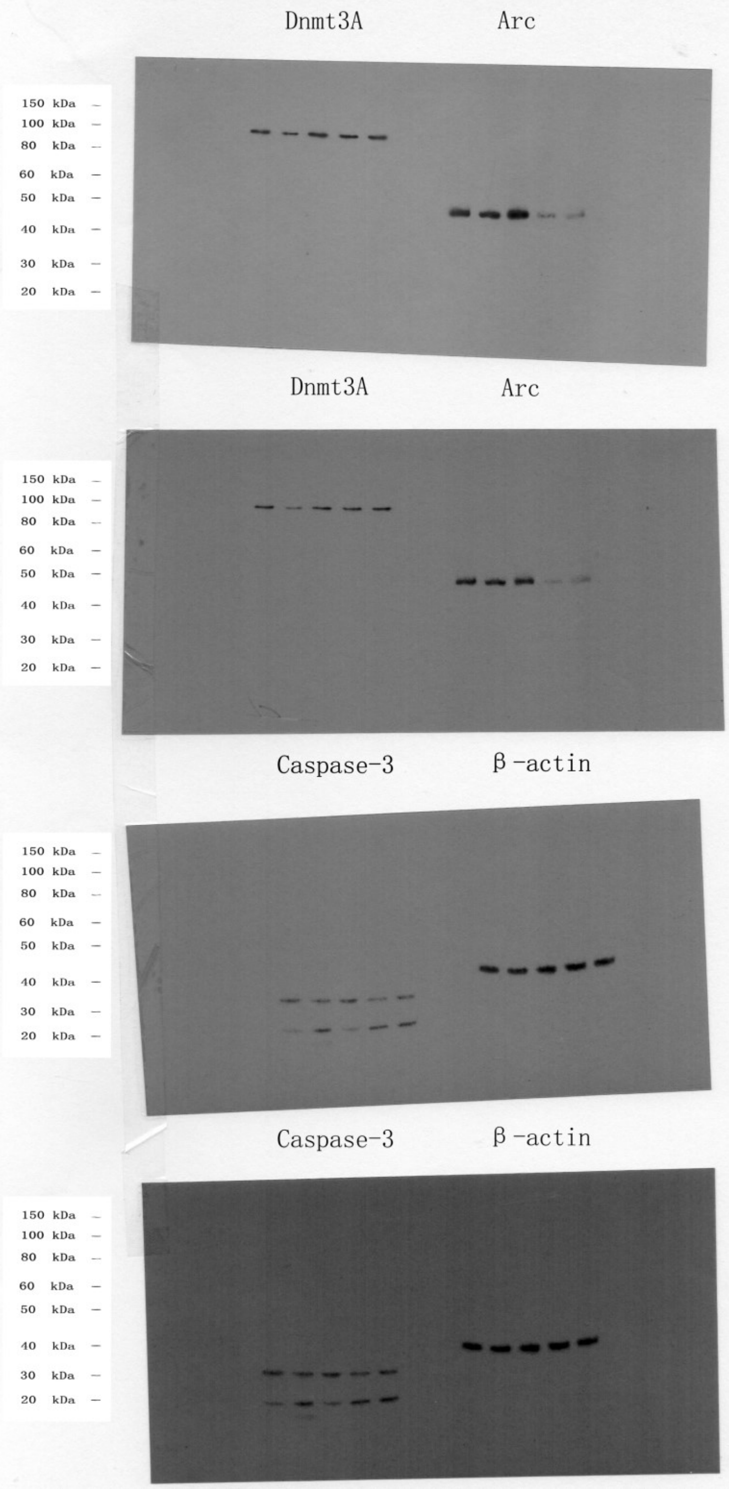
Dnmt3A
Arc
150 kDa
100 kDa
80 kDa
60 kDa
50 kDa
40 kDa
30 kDa
20 kDa
Dnmt3A
Arc
150 kDa
100 kDa
80 kDa
60 kDa
50 kDa
40 kDa
30 kDa
20 kDa
Caspase-3
β-actin
150 kDa
100 kDa
80 kDa
60 kDa
50 kDa
40 kDa
30 kDa
20 kDa
Caspase-3
β-actin
150 kDa
100 kDa
80 kDa
60 kDa
50 kDa
40 kDa
30 kDa
20 kDa
