# Supplementary material for: Dexmedetomidine reduces propofol-induced hippocampal neuron injury by modulating the miR-377-5p/Arc pathway
Source: BMC Pharmacol Toxicol. 2022 Mar 25;23:18. doi: 10.1186/s40360-022-00555-9 (PMC8957152; doi:10.1186/s40360-022-00555-9)
Supplement: Supplementary file 1 — Additional file 1. [file 40360_2022_555_MOESM1_ESM.pdf]

Figure S1. The binding site with the highest score between miR-377-5p and Arc 3'UTR predicted by the website.

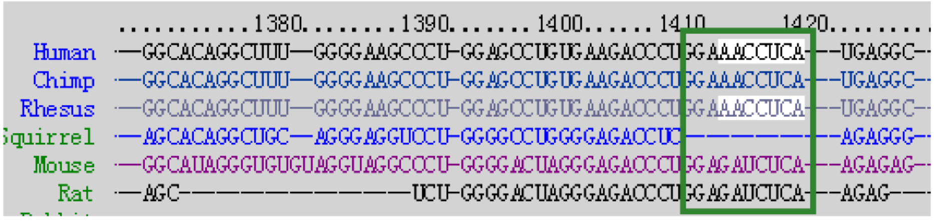

Arc 3'UTR 5'-GCTGCCGCCC**AGGGCAACC**GGCC-3'

Mmu-mir-377-5p 3'-CTTAAGTGGT**TCCCGTTGG**AGA-5'

	Predicted consequential pairing of target region (top) and miRNA (bottom)	Site type	Context++ score	Context++ score percentile	Weighted context++ score	Conserved branch length	P_{CT}
Position 436-442 of ARC 3' UTR<br>mmu-miR-377-5p	5' ...CAGAGAUGCUGAGGGAACCUCAA...<br>|||||| 3' CUUAAGUGGUUCCCGUUGGAGA	7mer-A1	-0.03	34	-0.03	0	N/A
Position 436-442 of ARC 3' UTR<br>mmu-miR-672-5p	5' ...CAGAGAUGCUGAGGGAACCUCAA...<br>|||||| 3' AGUGUGUGUCAUGUGGUUGGAGU	7mer-A1	-0.03	20	-0.01	0	N/A
Position 1273-1279 of ARC 3' UTR<br>mmu-miR-377-5p	5' ...UGAUUGCCCAGCUGAAACCUCAG...<br>||| |||||| 3' CUUAAGUGGUUCCCGUUGGAGA	7mer-A1	-0.08	57	-0.08	0.113	N/A

Poorly conserved

	Predicted consequential pairing of target region (top) and miRNA (bottom)	Site type	Context++ score	Context++ score percentile	Weighted context++ score	Conserved branch length	P_{CT}
Position 1235-1241 of ARC 3' UTR<br>rno-miR-377-5p	5' ...UGAUUGCCCAGCUGGAACCUCAU...<br>||| |||||| 3' CUUAAGUGGUUCCCGUUGGAGA	7mer-A1	-0.07	54	-0.07	0.113	N/A
Position 1235-1241 of ARC 3' UTR<br>rno-miR-672-5p	5' ...UGAUUGCCCAGCUGG--AACCUCAU...<br>||| |||||| 3' AGUGUGUGUCAUGUGGUUGGAGU	7mer-A1	-0.04	44	-0.04	0.113	N/A

Poorly conserved

	Predicted consequential pairing of target region (top) and miRNA (bottom)	Site type	Context++ score	Context++ score percentile	Weighted context++ score	Conserved branch length	P_{CT}
Position 137-143 of DLG4 3' UTR<br>rno-miR-194-3p	5' ...UCUAACUGGAUCGAGCCCACUGG...<br>||||||| 3' UCUAUUGUCGUCGGGUGACC	7mer-m8	-0.11	71	-0.11	0	N/A
Position 295-301 of DLG4 3' UTR<br>rno-miR-194-3p	5' ...CCAUGCACACAUAGACCCACUGG...<br>||| ||||||| 3' UCUAUUGUCGUCG-GGGUGACC	7mer-m8	-0.12	73	-0.12	0.091	N/A
